# Machine-learning classification identifies patients with early systemic sclerosis as abatacept responders via CD28 pathway modulation

**DOI:** 10.1172/jci.insight.155282

**Published:** 2022-12-22

**Authors:** Bhaven K. Mehta, Monica E. Espinoza, Jennifer M. Franks, Yiwei Yuan, Yue Wang, Tammara Wood, Johann E. Gudjonsson, Cathie Spino, David A. Fox, Dinesh Khanna, Michael L. Whitfield

**Affiliations:** 1 Department of Biomedical Data Science, Department of Molecular & Systems Biology, Geisel School of Medicine at Dartmouth, Lebanon, New Hampshire, USA.; 2 Department of Dermatology, Department of Medicine, Clinical Autoimmunity Center of Excellence and University of Michigan Scleroderma Program, University of Michigan, Ann Arbor, Michigan, USA.; 3 Department of Biostatistics, University of Michigan, Ann Arbor, Michigan, USA.; 4 Division of Rheumatology, Department of Medicine, Clinical Autoimmunity Center of Excellence and University of Michigan Scleroderma Program, University of Michigan, Ann Arbor, Michigan, USA.

**Keywords:** Autoimmunity, Clinical Trials, Expression profiling, Fibrosis, Skin

## Abstract

Here, the efficacy of abatacept in patients with early diffuse systemic sclerosis (dcSSc) was analyzed to test the hypothesis that patients in the inflammatory intrinsic subset would show the most significant clinical improvement. Eighty-four participants with dcSSc were randomized to receive abatacept or placebo for 12 months. RNA-Seq was performed on 233 skin paired biopsies at baseline and at 3 and 6 months. Improvement was defined as a 5-point or more than 20% change in modified Rodnan skin score (mRSS) between baseline and 12 months. Samples were assigned to intrinsic gene expression subsets (inflammatory, fibroproliferative, or normal-like subsets). In the abatacept arm, change in mRSS was most pronounced for the inflammatory and normal-like subsets relative to the placebo subset. Gene expression for participants on placebo remained in the original molecular subset, whereas inflammatory participants treated with abatacept had gene expression that moved toward the normal-like subset. The Costimulation of the CD28 Family Reactome Pathway decreased in patients who improved on abatacept and was specific to the inflammatory subset. Patients in the inflammatory subset had elevation of the Costimulation of the CD28 Family pathway at baseline relative to that of participants in the fibroproliferative and normal-like subsets. There was a correlation between improved *Δ*mRSS and baseline expression of the Costimulation of the CD28 Family pathway. This study provides an example of precision medicine in systemic sclerosis clinical trials.

## Introduction

Systemic sclerosis (SSc) is a rare, progressive autoimmune disease of unknown etiology; it is characterized by extracellular matrix deposition as well as vascular and immunologic abnormalities (1, 2). Patient heterogeneity is an inherent feature of SSc, as manifested by variable skin fibrosis, autoantibodies, organ involvement, and progression. There are two reported clinical subtypes defined by the extent of skin involvement: diffuse cutaneous SSc (dcSSc) and limited cutaneous SSc. This study is focused on patients with dcSSc.

Four intrinsic molecular subsets that break down this heterogeneity have been identified across multiple cohorts and organ systems using whole-genome expression profiling (3–6). These include the inflammatory subset, which is characterized by increased immune and fibrotic processes; the fibroproliferative subset, with increased fibrotic, immune, and cell-cycle-related processes; the normal-like subset, with gene expression patterns that more closely resemble those of healthy controls; and finally, the limited subset, which primarily comprised patients with limited cutaneous SSc. Gene expression differences observed between patients may lead to the differential response to therapies. When results from clinical trials are analyzed in aggregate without considering molecular subsets, the differing responses may lead to a report of overall therapy failure; however, retrospective data analyses identified that some molecular SSc subsets respond better to particular drugs (7). These data suggest that molecular intrinsic subsets should be taken into consideration prior to clinical trials. This marks an important step toward precision medicine in SSc.

Here, we report a detailed analysis of gene expression data from skin biopsies from patients treated with abatacept, which is a fusion protein that comprises the extracellular domain of CTLA-4 and the Fc region of IgG1. Abatacept binds to CD80/86 and outcompetes CD28 binding, thus preventing T cell activation (8). A pilot study of 8 participants with SSc in a 24-week, placebo-controlled trial identified key changes in patients who improved on abatacept, in comparison with those who did not (9). The study demonstrated a reduction in the inflammatory signature and CD28-dependent signaling, suggesting that abatacept may be more beneficial to patients with SSc who are in the inflammatory molecular subset.

A subsequent randomized, double-blind, placebo-control, phase II trial of 88 participants, the Abatacept in Systemic SclErosis Trial (ASSET), showed that while abatacept was well tolerated, the change in modified Rodnan skin score (mRSS) in the abatacept group was greater but not statistically significant compared with that in the placebo group (10). The data from this study showed that patients from the abatacept treatment group who were classified into the inflammatory intrinsic subset at baseline showed the largest and a statistically significant decrease in mRSS in comparison with that of patients in the placebo treatment group.

Here, we identified gene expression changes in skin occurring in the abatacept phase II clinical trial that related to the clinical improvement in patients exhibiting an elevated inflammatory molecular signature at baseline. We found that patients in the inflammatory intrinsic gene expression subset had higher expression of the Costimulation of the CD28 Family Reactome Pathway, a signature related to abatacept’s mechanism of action, when compared with that of patients in the fibroproliferative or normal-like subsets. Furthermore, though the expression of this pathway decreased in all patients on abatacept, the inflammatory patient subset was the only population that demonstrated a statistically significant decrease in response to abatacept treatment. We also found that the baseline expression of Costimulation of the CD28 Family pathway was significantly correlated to changes in mRSS in patients in the inflammatory subset. Gene expression in the skin of individuals in the inflammatory and fibroproliferative subsets moved toward the normal-like subset, whereas gene expression in the skin of individuals in the placebo arm largely remained in the molecular subtype they were in at baseline. We therefore conclude that inflammatory patients have elevated expression of pathways targeted by abatacept, which decreases upon treatment, and these patients are thus most likely to clinically respond to this therapy.

## Results

### Clinical and demographic characteristics of participants.

Eligible participants with early dcSSc (≤3 years from onset of first non–Raynaud’s sign or symptom) were randomized in a 1:1 ratio to either abatacept (125 mg subcutaneous) or matching placebo, stratified by duration of dcSSc. The coprimary endpoints were change in mRSS and safety over 12 months. Escape therapy with immunomodulatory agents was permitted as an add-on therapy to study medications taken due to worsening of dcSSc starting at month 6 (10). The decision to initiate escape therapy was based on investigator discretion. No biologic agents were allowed as escape therapy.

Abatacept was well tolerated. Among the 88 participants (44 in each treatment group), the adjusted mean change in mRSS at 12 months was –6.24 in the abatacept group and –4.49 in the placebo group, with a least-squares mean treatment difference of –1.75 (95% CI, –4.93, 1.43; *P* = 0.28) and marked individual variability (10). Secondary efficacy outcome measures were statistically significant, favoring abatacept.

Skin biopsies were collected from 84 of the 88 patients enrolled in the ASSET clinical trial at baseline and at 3 and 6 months. The RNA was prepared and sequenced by Illumina RNA-Seq. Sample reads were parsed for quality, repeats, and missing information, resulting in 140 biospecimens that were used in the initial analysis (Supplemental Figure 3; supplemental material available online with this article; https://doi.org/10.1172/jci.insight.155282DS1). Data cleaning was done stepwise. First, duplicate patient samples, those with low-quality sequence reads, and those missing either their baseline or 6-month biopsies were removed (level 1, representing 36 skin biopsies from 20 participants). In order to allow clinical improvement to be determined accurately, patients who escaped prior to 12 months or had missing mRSS data at 12 months were also removed (level 2 filtering, representing 57 skin biopsies from 21 participants). The remaining 140 skin biopsies from 47 patients were analyzed (Supplemental Figure 3). Improvement was classified by patients who had a reduction in their baseline mRSS by 20% or 5 points. There were no significant differences between the abatacept and placebo arms after biospecimen curation with regards to clinical characteristics, including sex, age, and race (Table 1). Key analyses were subsequently repeated on the larger set of 197 biospecimens from 68 patients who passed level 1 filtering.

### Gene expression in baseline skin biopsies is associated with molecular subsets.

**Figure 1:**
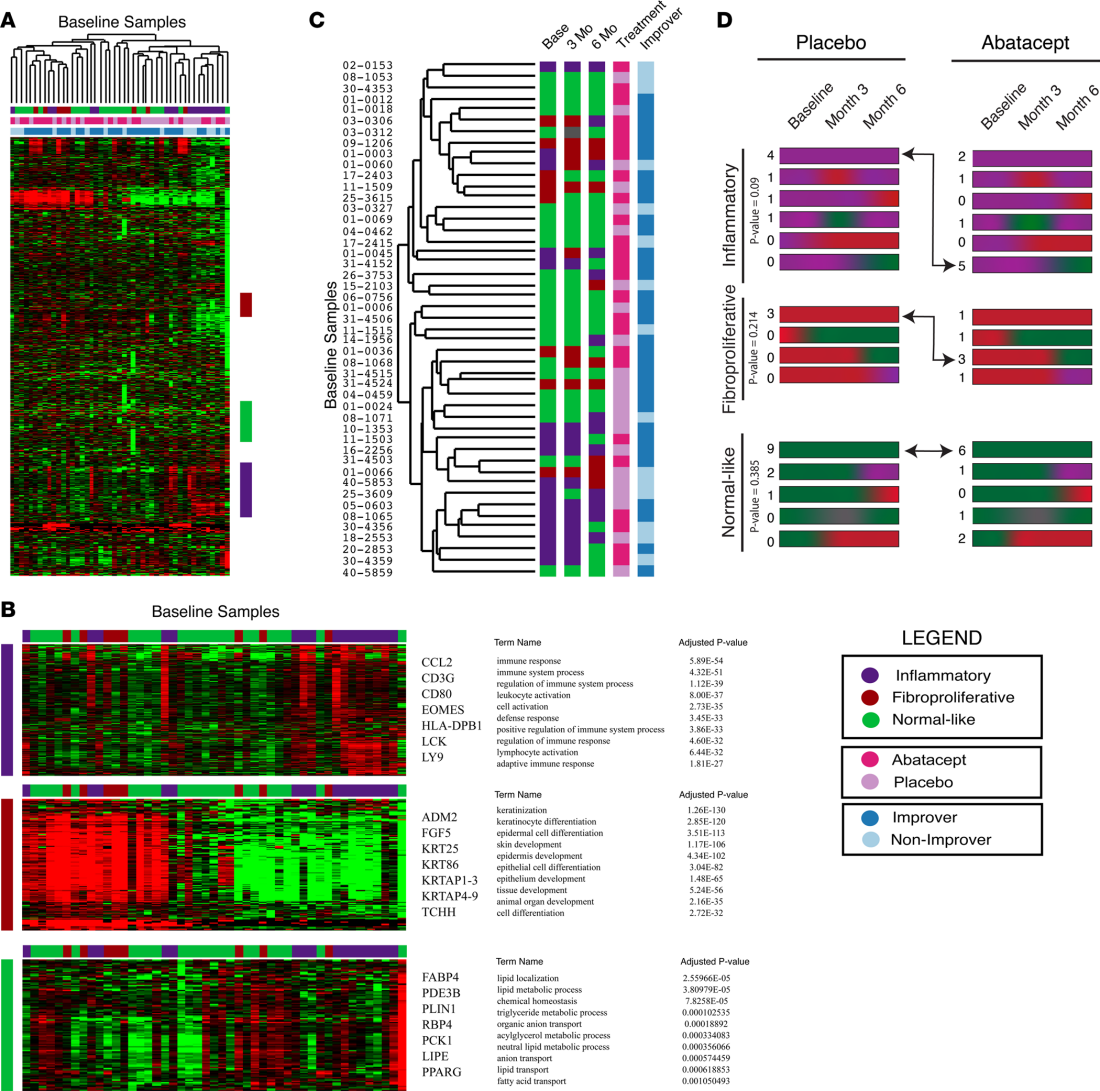
Baseline skin biopsies recapitulate intrinsic subset biology. (**A**) Hierarchical clustering of log_2_ median-centered data, with the most variable genes (2,179 genes) from a 2-fold, 2-array heatmap using Cluster 3.0. Color bars represent intrinsic subset by SVM classifier and treatment-improvement status. (**B**) Expansion of gene clusters from **A** that correlate with intrinsic subset SVM call. Selected genes are shown. The top 10 most significant pathways (FDR < 5%) from g:Profiler are shown. (**C**) Dendrogram with color bars for SVM calls at baseline and in 3- and 6-month skin biopsies. Abatacept and placebo treatment is shown on the far right, color-coded by improver and nonimprover status. (**D**) Progression of subtypes from baseline to month 6. Numbers of individuals with each progression are shown to the right.

Analyses of baseline skin biopsies recapitulated the previously defined molecular subsets (Figure 1A). Assessment of batch effect was performed on raw counts of RNA-Seq transcripts with the gPCA package in R, which revealed no detectable differences due to batch (Supplemental Figure 4; *P* = 0.612). Similar analyses for batch effect were performed after reads per kb of transcript per million mapped reads (RPKM) normalization, which similarly revealed no significant variation explained by batch (Supplemental Figure 5; *P* = 0.594). Genes and pathways associated with molecular subset calls assigned using the previously trained support vector machine (SVM) classifier (11) are show in Figure 1B (10). We found that the patients assigned to the inflammatory subset had an enrichment in pathways consistent with an active immune response, patients assigned to the fibroproliferative subset had active pathways related to cell differentiation and keratinization, and patients assigned to the normal-like subset had increased expression of pathways for lipid-associated processes. Of note, the hierarchical clustering of baseline samples did not show patterns in clustering based on treatment arm (abatacept or placebo) or improvement status (Figure 1C). The main driver of the clustering of baseline samples is intrinsic molecular subtype.

The genes and pathways with increased expression in the inflammatory molecular subset (Figure 1B) showed enrichment in key immune-related genes that are implicated in abatacept’s mechanism of action (e.g., CD80). These data are consistent with those of the pilot study, which suggested that the inflammatory subset might be most responsive to treatment, encouraging further analysis (9).

### Change in subtype over time between treatment arms.

In order to understand if patients in the inflammatory or fibroproliferative subsets become more normal like over time, we analyzed the SVM subtype classifications at baseline and at 3 months and 6 months after treatment (Figure 1D). We observed that the molecular subsets to which the samples were assigned were largely stable over time when considering biopsies from the same patient, with 30 of 47 (63%) participant molecular subsets remaining unchanged (Figure 1D). Participants in the abatacept arm that were in the inflammatory subset demonstrated a shift toward a more normal-like signature, with 5 of 9 (56%) participants shifting from inflammatory to normal-like and 3 of 9 (33%) remaining in the inflammatory subset. In contrast, 6 of 7 (86%) participants who were inflammatory at baseline in the placebo arm were also inflammatory at their 6-month time point (Figure 1D; inflammatory, Fisher’s exact test, *P* = 0.09). Of the 6 participants classified as fibroproliferative in abatacept arm, 4 of 6 (67%) were normal like at 6 months, while 1 remained fibroproliferative and 1 was classified as inflammatory. In contrast, of the 3 participants classified as fibroproliferative at baseline in the placebo arm, all remained in that subset at 3 and 6 months (Figure 1D). Although there was an increase in the number of patients in the fibroproliferative subset that changed to the normal-like subset on abatacept treatment relative to placebo treatment, these differences did not reach statistical significance (Figure 1D; fibroproliferative, Fisher’s exact test, *P* = 0.214). Participants that were normal-like at baseline did not show a significant change in subtype, regardless of treatment (Figure 1D and Supplemental Table 5; normal like, Fisher’s exact test; *P* = 0.385). Interestingly, a small subset of patients in the normal-like subset became inflammatory (3 participants) or fibroproliferative (3 participants) at 6 months. Therefore, this suggests that some number of patients may move from the normal-like subset back to a more pathogenic gene expression signature.

### GSEA reveals therapeutic modulation of pathways consistent with abatacept treatment.

Gene set enrichment analysis (GSEA) was performed to interrogate and compare enriched pathways across baseline and 6-month biopsies in response to treatment. We performed GSEA on all participant biopsy gene expression profiles and parsed by treatment and improvement status to identify potential associations between therapy-mediated improvement and molecular signatures. As a result, there were 64 pathways enriched in baseline biopsies and 2 pathways enriched in 6-month time point biospecimens. We found that immune-related pathways are enriched in the baseline biopsies of participants who improved (improvers) on abatacept but not in 6-month samples, suggesting that these pathways have decreased in the 6-month biopsies (Supplemental Table 1). As in the pilot investigation (9), we identified the Costimulation of the CD28 Family pathway as one of the top pathways enriched in baseline biopsies (0.006% FDR) and subsequently modulated by abatacept in improvers, as demonstrated by the directionality of regulation (Table 2). The FDR acts as a contextualizing statistic, reporting the chances of false discovery (which we prefer to be low) of the genes enriched in this pathway. We did not find this pathway enriched at baseline in abatacept-treated participants who did not improve (nonimprovers) (Supplemental Table 1C), placebo-treated improvers (Supplemental Table 1B), or placebo-treated nonimprovers (Supplemental Table 1D). These data suggest that only in improvers on abatacept do we see molecular modulation of pathways directly linked to abatacept’s mechanism of action.

Analysis of ASSET clinical outcomes showed that the inflammatory subset of patients showed the largest clinical improvement, as determined by a decrease in mRSS (10). Accordingly, we divided our data set by intrinsic subset to identify molecular pathways specific to the subsets in the GSEA analysis. The top 10 pathways for inflammatory, fibroproliferative, and normal-like subsets can be found in Supplemental Table 2, Supplemental Table 3, and Supplemental Table 4, respectively. Importantly, we found that, in patients in the inflammatory subset that improved on abatacept, there was an enrichment of the Costimulation of the CD28 Family pathway in the pretreatment biopsies (Table 2). This pathway was not significantly changed in patients on placebo or patients in the fibroproliferative subset treated with abatacept (Table 2). Interestingly, we saw that nonimproving patients in the inflammatory subset on abatacept still showed modulation of the Costimulation of the CD28 Family pathway, albeit at the lower pathway rank (Table 2). This suggests that while these patients might not be clinically improving, we still observed effective targeting of the pathway at the molecular level. Taken together, these data demonstrate a measurable decrease in pathways related to abatacept treatment that was specific to patients in the inflammatory subset. The change in pathway expression occurred regardless of clinical improvement, as shown through the enrichment of therapy-relevant pathways in baseline samples from a specific molecular subset of patients. It also reinforced the application of precision medicine to identify patients who could potentially benefit the most from a specific therapy.

### Costimulation of the CD28 Family pathway is elevated in patients in the inflammatory subset and decreases when patients are treated with abatacept.

**Figure 2:**
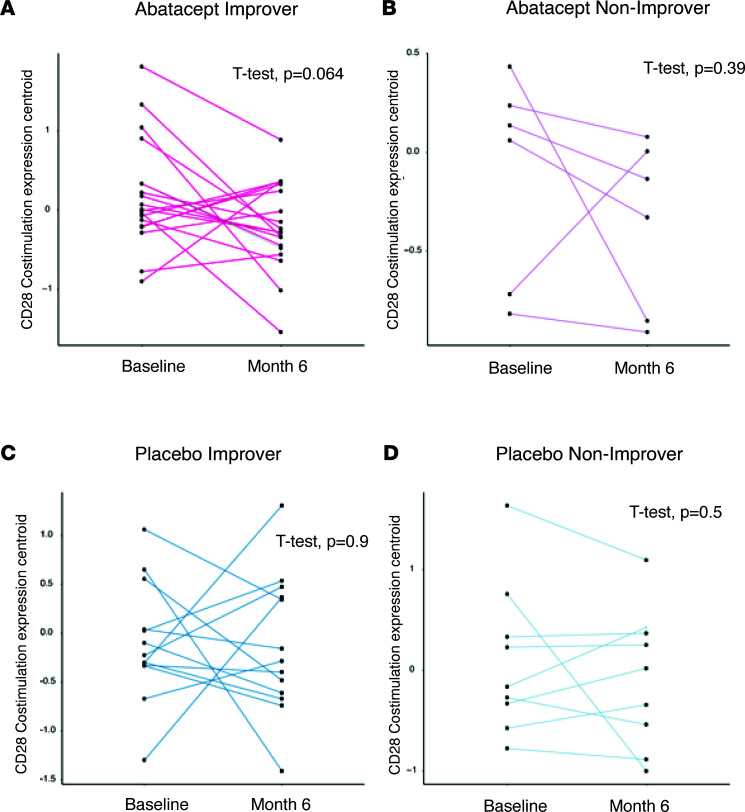
Comparison of the Costimulation of the CD28 Family average expression for patients at baseline and 6-month time points. (**A**) Average gene expression for core enrichment genes in the Costimulation of the CD28 Family pathway in patients on abatacept who improved. Data are log_2_ and median centered. (**B**) Average gene expression for core enrichment genes in the Costimulation of the CD28 Family pathway in patients on abatacept who did not improve. Data are log_2_ and median centered. (**C**) Average gene expression for core enrichment genes in the Costimulation of the CD28 Family pathway in patients on placebo who improved. Data are log_2_ and median centered. (**D**) Average gene expression for core enrichment genes in the Costimulation of the CD28 Family pathway in patients on placebo who did not improve. Data are log_2_ and median centered. Paired *t* test *P* values are shown.

With Costimulation of the CD28 Family being implicated in the pilot study (9) and in this work, we sought to take a more granular look and investigate the expression trends of the genes driving the enrichment of this pathway. We found that the core enrichment genes (*n* = 23) showed a decreasing trend between baseline and 6-month time points in improvers treated with abatacept (Figure 2A, *P* = 0.064), with no significant changes in abatacept-treated nonimprovers (Figure 2B), placebo-treated improvers (Figure 2C), or placebo-treated nonimprovers (Figure 2D).

**Figure 3:**
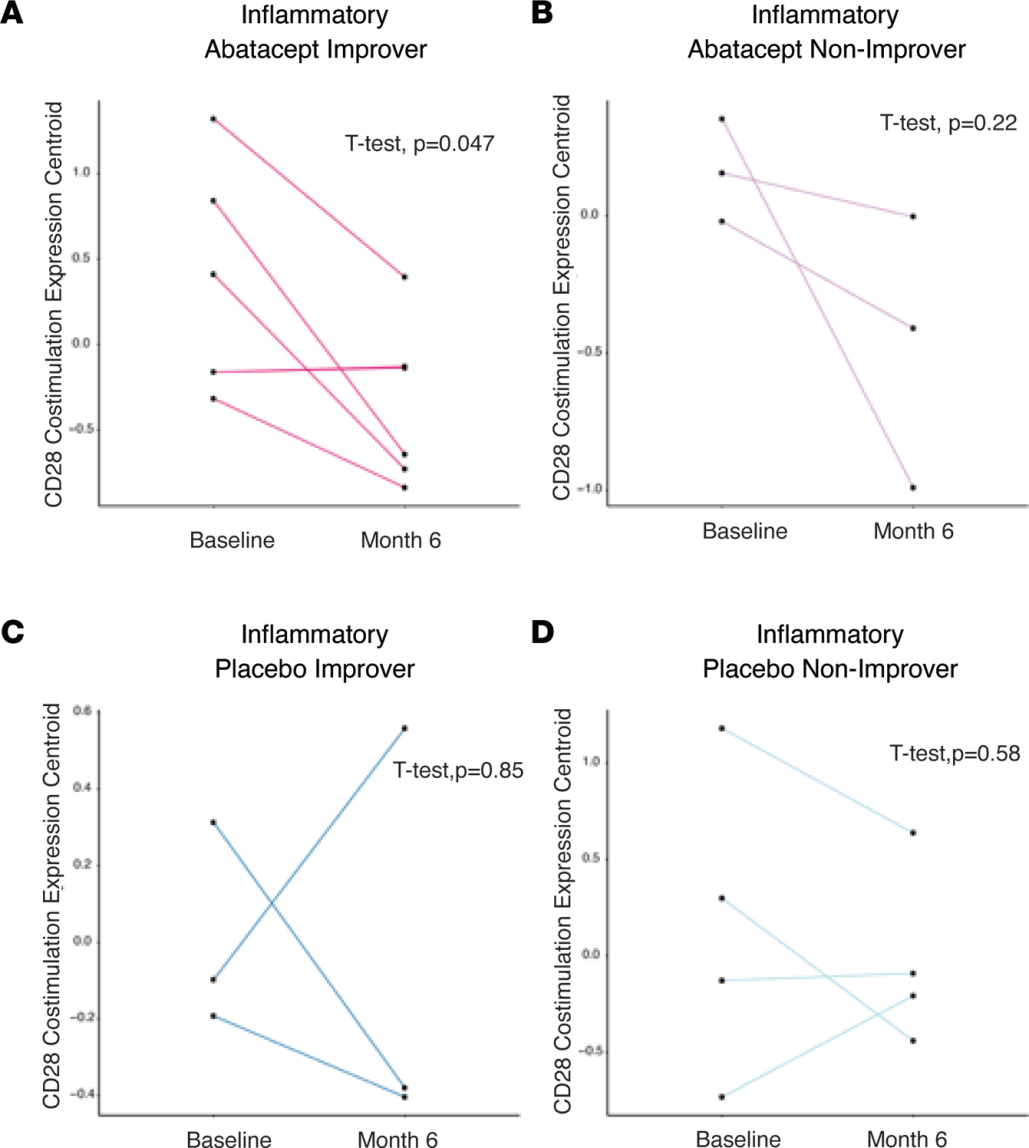
Comparison of the average expression of the Costimulation of the CD28 Family pathway in the inflammatory subset at baseline and 6-month time points. (**A**) Average gene expression for core enrichment genes in the Costimulation of the CD28 Family pathway in patients in the inflammatory subset on abatacept who improved. Data are log_2_ and median centered. (**B**) Average expression for core enrichment genes of the CD28 pathway in patients in the inflammatory subset on abatacept who did not improve. Data are log_2_ and median centered. (**C**) Average gene expression for core enrichment genes in the Costimulation of the CD28 Family pathway in patients in the inflammatory subset on placebo who improved. Data are log_2_ and median centered. (**D**) Average gene expression for core enrichment genes in the Costimulation of the CD28 Family pathway in patients in the inflammatory subset on placebo who did not improve. Data are log_2_ and median centered. Paired *t* test *P* values are shown.

This trend was further clarified when the baseline biopsies were stratified by intrinsic subset. While 8 of 9 patients in the inflammatory subset on abatacept showed a decrease in the expression of the core enrichment genes between baseline and 6-month time points (Figure 3, A and B), only the improvers showed a significant decrease (Figure 3A, *P* = 0.047). Patients in the inflammatory subset on placebo did not show significant changes in the core enrichment genes from Costimulation of the CD28 Family (Figure 3, C and D). Of note, this pathway did not show any significant change in patients in the fibroproliferative (Supplemental Figure 1) or normal-like subsets (Supplemental Figure 2).

**Figure 4:**
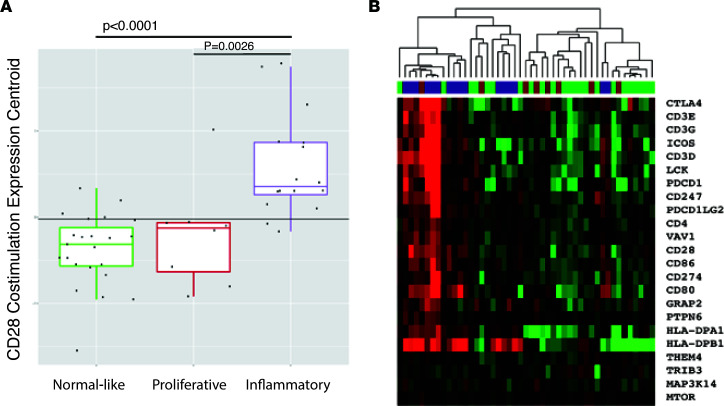
Baseline skin biopsies classified as the inflammatory intrinsic subset have elevated expression of genes that are enriched in response to CD28 costimulation. (**A**) Baseline skin biopsies (*n* = 47) were log_2_ and median centered. Means were found to be significantly different (*P* < 0.001, ANOVA). Tukey’s test for multiple comparisons *P* values are shown. (**B**) Hierarchical clustering of core enrichment genes from the Costimulation of the CD28 Family and baseline biospecimens**.**

Unsurprisingly, at baseline, the inflammatory subset of patients had significantly higher expression of genes that are enriched in response to CD28 costimulation, compared with the baseline expression of this pathway in patients who were in the fibroproliferative (*P* = 0.0026) or normal-like subsets (Figure 4, *P* < 0.0001). We repeated this analysis, adding back the patients removed by level 2 filtering and found that this difference remained when the set of 197 skin biopsies (68 patients) was analyzed (Supplemental Figure 7). Thus, we postulate that patients in the inflammatory subset are more likely to improve on abatacept, because genes and pathways relevant to its mechanism of action have increased expression at baseline, and, thus, patients in the inflammatory subset are more responsive to therapeutic modulation by abatacept.

### Expression of Costimulation of the CD28 Family at baseline in patients in the inflammatory subset is significantly related to change in skin thickness score at 12 months.

**Figure 5:**
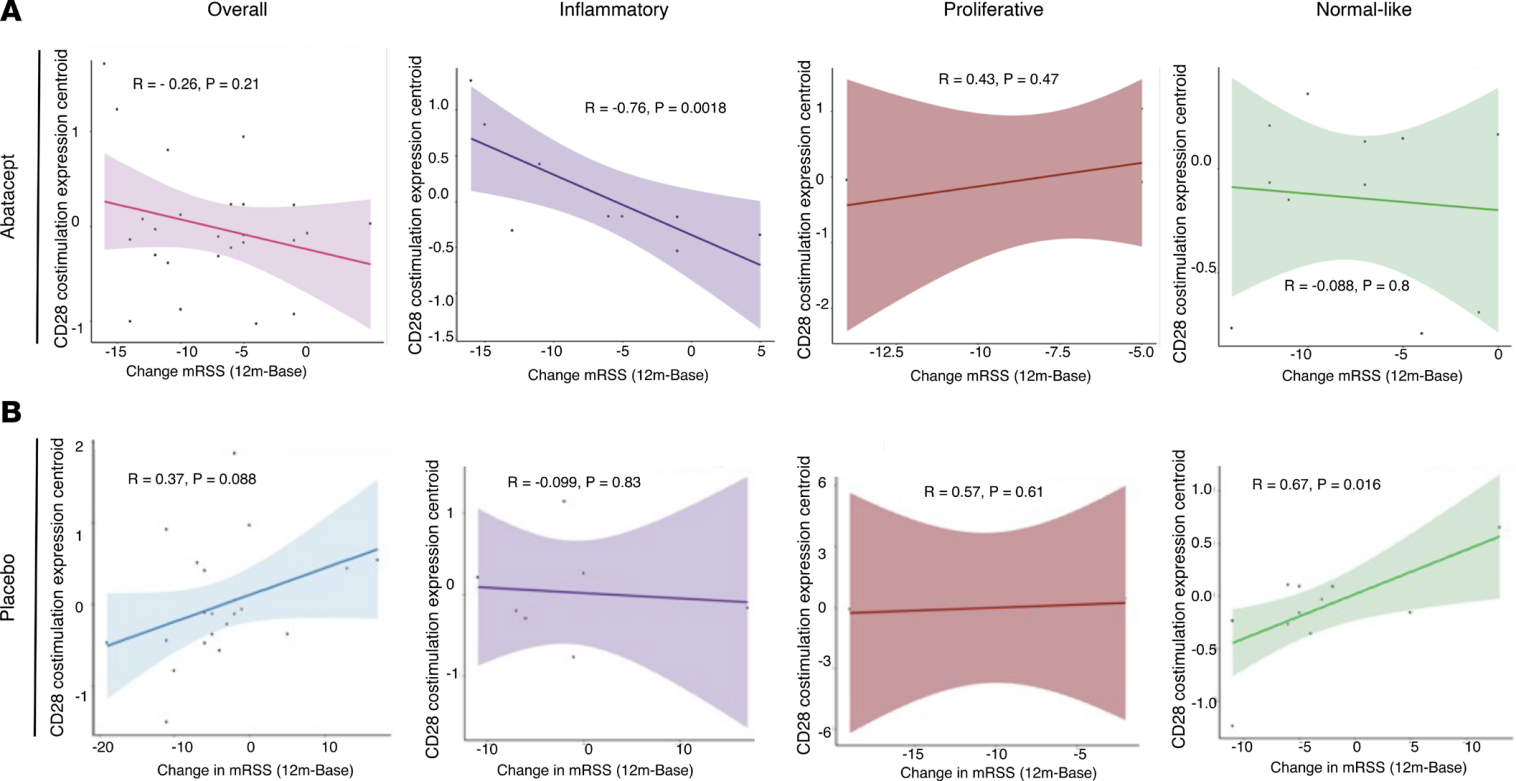
Baseline expression of genes that are enriched in response to CD28 costimulation is associated with patient improvement on abatacept and on placebo therapy in the inflammatory subset. (**A**) All patients on abatacept (*n* = 25) divided by intrinsic subset: inflammatory, fibroproliferative, and normal like. (**B**) All patients on placebo (*n* = 22) divided by intrinsic subset: inflammatory, fibroproliferative, and normal like.

To determine whether the Costimulation of the CD28 Family signature was associated with clinical improvement on abatacept, we calculated change in mRSS correlations between baseline and 12 months and the original baseline expression. When considering the 47 patients who passed filtering, there were no significant correlations in the abatacept (Figure 5A) or placebo (Figure 5B) arms between baseline mRSS and total change across time points. In conducting this analysis parsed by molecular subtype, we observed significant correlation between baseline expression of core enrichment genes of the Costimulation of the CD28 Family pathway and decrease in mRSS for patients in the inflammatory subset on abatacept (Figure 5A, Pearson’s, *r* = –0.76, *P* = 0.018). This correlation was not significant for patients in the inflammatory subset on placebo (Figure 5B), patients in the fibroproliferative subset on abatacept (Figure 5A) or placebo (Figure 5B), or patients in the normal-like subset on abatacept (Figure 5A). Conversely, we observed the opposite association between change in mRSS and patients in the normal-like subset on placebo (Figure 5B, Pearson’s, *r* = 0.67, *P* = 0.016). We repeated this analysis in the 68 patients obtained when only level 1 filtering was applied and confirmed the result in the larger cohort (Supplemental Figure 8).

### Inclusion of all patients in the inflammatory subset at baseline is significantly related to the expression of the Costimulation of the CD28 Family.

**Figure 6:**
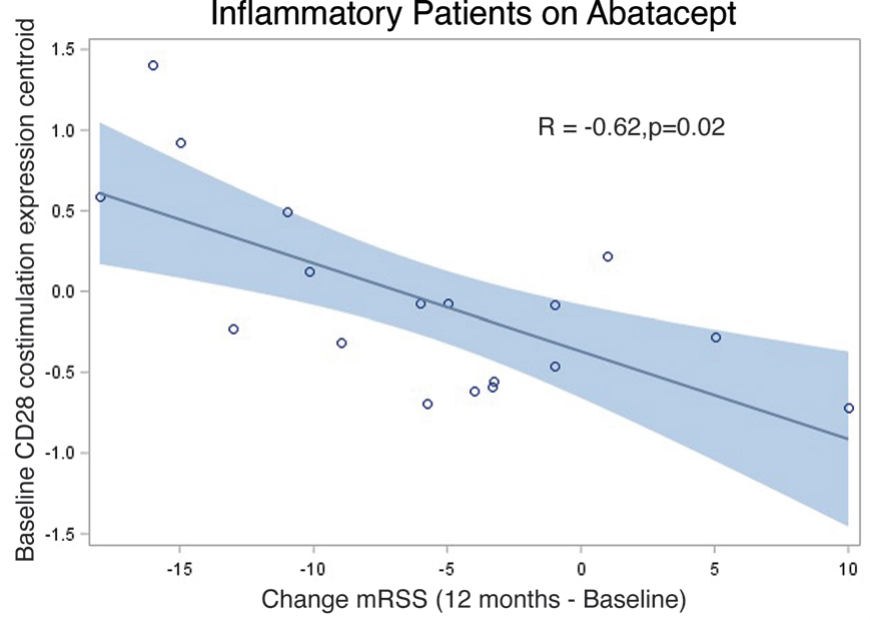
Baseline expression of genes that are enriched in response to CD28 costimulation is associated with estimated change in skin severity on abatacept in the inflammatory subset. A Pearson’s correlation *P* value is shown (*n* = 18).

To identify pathways modulated between baseline and after treatment time points, prior analyses required removal of samples that were missing RNA-Seq measurements or clinical data at the time point after treatment (level 1 and 2 filtering, Supplemental Figure 3). However, using only baseline gene expression for subsequent analyses means that patients for whom RNA-Seq data were missing can now be added to analyses and in addition, linear-mixed models can be used to estimate mRSS in time points after treatment for patients who escaped therapy (10). We found that our results remained true when all patients in the inflammatory subset on abatacept (regardless of filtering) in the trial were considered (Figure 6, Pearson’s, *r* = –0.62, *P* = 0.02) relative to change in MRSS from baseline to 12 months. We also analyzed the correlation between average expression centroid of the core enrichment genes from the Costimulation of the CD28 Family pathway in the 6-month biopsy and the change in mRSS from baseline to 6 months. We found that the overall relationship holds at the 6-month time point, although the slope of the line decreased, likely reflecting the treatment-associated decrease in the pathway expression (Supplemental Figure 6, A and B).

## Discussion

The rarity of and heterogeneity in SSc often lead to statistically underpowered clinical trials that result in outcomes that are difficult to interpret. Retrospective analyses of clinical trials have previously suggested that assessing patient heterogeneity can help identify patients who are most likely to benefit from specific therapies. Gene expression analyses of a pilot study of abatacept suggested that inflammatory pathways were highly expressed in patients who improved and that the expression of these pathways decreased with improvement (9). A meta-analysis of clinical trials in SSc came to a similar conclusion using data from 5 clinical studies, including mycophenolate mofetil, rituximab, abatacept, nilotinib, and fresolimumab. This study showed that abrogation of inflammatory pathways, regardless of treatment, was most significantly associated with clinical improvement (7). This meta-analysis also showed that patients with a high TGF-β signature at baseline were most likely to benefit in a trial of fresolimumab, which targets TGF-β1 (7).

In this work, we analyzed the gene expression at baseline of patients in the abatacept clinical trial and its changes with clinical improvement across molecular subtypes in the context of therapy. Consistent with results of previous work (9), we found that patients with SSc in the inflammatory subset had an increased expression of pathways targeted by abatacept at baseline and were the most likely group to benefit from treatment. We found that the Costimulation of the CD28 Family pathway, which is directly related to mechanism of action of abatacept, was elevated in patients in the inflammatory subset at baseline and that the decrease in expression of this pathway correlated with clinical improvement and resolution of skin fibrosis, as represented by a decrease in skin severity score. Interestingly, in the placebo arm, patients in the normal-like subset seemed to worsen if they had high expression of CD28 family genes at baseline. While these data may be explained by a select few samples, we note that patients in the normal-like subset who improved on abatacept showed modulation of Costimulation of the CD28 Family and that the expression of these genes seemed to decrease in most patients in the normal-like subset treated with the drug. As with the clinical study (10), we see no clear effect on the fibroproliferative subset of patients on abatacept, except for a nonstatistically significant increase in the number of patients in the fibroproliferative subset who move to the normal-like subset.

These data suggest that intrinsic subsets may provide a powerful platform to stratify the patient population with SSc prior to assessing the clinical efficacy of a therapeutic agent. In participants treated with abatacept who showed clinical improvement, we observed a consistent and statistically significant shift from the inflammatory to the normal-like subset by month 6. Interestingly, a similar shift was observed for a subset of fibroproliferative participants, but the change did not reach statistical significance. Participants in the placebo arm of the study were more likely to maintain a stable subset assignment from baseline to 6 months, although we note several exceptions in the inflammatory subset that include 1 biopsy classified as fibroproliferative at the 3-month time point and 1 as normal like at the 3-month time point, both of which were bounded by inflammatory subset assignments at baseline and 6 months. One patient was observed to shift from inflammatory to fibroproliferative at 6 months. These changes represent 3 biopsies of 21 (14%) for the patients in the inflammatory subset in the placebo arm, which is within the 15% classification error rate (11). Individuals in the placebo arm predominantly remained in their baseline molecular subtype, largely unchanged, reproducing prior observations (4), suggesting that the subtypes are stable through 6 months in the absence of disease-modifying treatment. These data provide an approach by which we can use precision medicine in SSc to identify those patients most likely improve on therapy, determine target engagement, and track an individuals’ molecular changes in disease state over time. This and our previously published analyses (3–5, 7, 11) suggest that clinical trialists should acknowledge molecular heterogeneity in early SSc and should enrich for pharmacologic target–specific subset (such as inflammatory intrinsic subset for abatacept, mycophenolate mofetil, etc.) or stratify randomization based on these subsets. In addition, our recently published meta-analysis highlights the role of scleroderma autoantibodies as an enrichment criterion and the overlap with the intrinsic gene expression sets (12, 13).

This study has a number of limitations. RNA-Seq of skin biopsies from participants followed by subset classification using our machine-learning algorithm confirmed the a priori hypothesis that the inflammatory subset would improve. Although this is very promising, this result needs to be confirmed in a prospective phase III clinical trial. The study population remains relatively small, and these results need to be confirmed in a larger cohort of patients. Some individuals escaped therapy due to clinically worsening disease during the trial, including mRSS increase, pulmonary hypertension, or renal crisis, which could potentially introduce biases into our analyses. Those that escaped therapy included 10 individuals in the inflammatory subset, which could reduce the generalizability of our findings.

While the use of RNA-Seq to further investigate the tissue biology of these individuals brought many benefits, the filtering for data quality and escape events resulted in a reduction in samples analyzed here. We addressed this by adding back samples that were removed in level 2 filtering and repeating key analyses. We found that the exclusions increased the signal-to-noise ratio but did not change the main findings of the analysis, as similar trends in Costimulation of the CD28 Family pathway expression among molecular subtypes were still observed (Supplemental Figure 7), with the highest expression difference noted among participants in the inflammatory subset (*P* < 0.05). Correlations between the Costimulation of the CD28 Family pathway and mRSS demonstrated the same trends when all biopsies were added back in from those individuals who were removed in level 2 filtering (Supplemental Figure 8).

In summary, we demonstrated that measuring molecular heterogeneity in patients with SSc can assist in the interpretation of clinical trial results and may be valuable tool that can be used in future studies to identify the patients most likely to improve on a given therapy.

## Methods

### Participants in the ASSET clinical trial.

Participants in this study were from a phase II, investigator-initiated, randomized, double-blind, placebo-controlled trial of abatacept in patients with early-stage dcSSc, defined as disease duration of less than 36 months at enrollment (Clinicaltrials.gov NCT02161406). dcSSc was defined as skin thickening, proximal as well as distal, to the elbows or knees with or without involvement of the face and neck. Study participants were treated for 12 months on double-blind study medication and were offered an additional 6 months of open-label s.c. abatacept therapy as previously described (14). Key inclusion criteria and have been described in the clinical study (10). Oral corticosteroids (≤10 mg/d prednisone or equivalent) and NSAIDs were permitted if the patient was on a stable dose regimen for equal to or more than 2 weeks prior to and including the baseline visit, but no background immunomodulatory therapies were allowed. Improvement was defined as a 5 point or greater than 20% change in mRSS between baseline and 12 months, as used in the primary ASSET clinical trial analyses (10, 15).

### Collection and RNA-Seq.

Skin biopsies were collected (3 mm) from the forearm of participants enrolled in the trial. Biopsies were collected at baseline, 3 months, and 6 months. Samples were stored in RNA*later* (Thermo Fisher Scientific) and subsequently homogenized using a Qiagen Tissuelyser II. RNA was purified using Qiagen’s RNeasy Fibrous Tissue minikit. Quality and concentration of RNA were assessed on a TapeStation 4400 (Agilent). 100 ng total RNA was used for library preparation using the TruSeq Stranded Total RNA Library Prep Kit (Illumina). Libraries were prepared manually or were automated on an epMotion 5075t (Eppendorf). Libraries were assessed for size and concentration using the TapeStation 4400. Sequencing libraries were quantified on a Qubit fluorometer 3.0 (Thermo Fisher Scientific) prior to normalization for equimolar pooling. Single-index paired-end sequencing was performed on Illumina’s NextSeq 500 to achieve more than 40 million reads per sample. Sequencing runs for samples were included in or excluded from analyses according to experimental and patient-specific factors outlined in Supplemental Figure 3 (i.e., level 1 and level 2 filtering as outlined).

### Subset classification.

RNA-Seq data were first normalized using feature-specific quantile normalization (16), which allowed subset classification. Molecular subset classifications assigned in the ASSET phase II clinical trial paper were used for these analyses (10). The SVM classifier used to assign SSc intrinsic molecular subsets was previously developed and its construction (including cross-validation of training and test sets), internal, and external validation in independent data sets is described in Franks et al. (11).

### Data processing and visualization.

233 biospecimens were prepared and sequenced. A custom RNA-Seq pipeline was applied to align the paired-end sequencing data. First, we applied the cutadapt (ref. 17; https://cutadapt.readthedocs.io/en/stable/) (version 1.15) to remove the adapter sequence (in our case it was “AGATCGGAAGAGC”) for the FASTQ files. Then, STAR (version 2.5.3a) was applied to align the reads to the hg19 human genome (18). Next, gene abundance was quantified using RSEM (version 3.3.9) (19). Finally, normalized RPKM was calculated using the TMM function (edgeR package) (20). The complete data set is available on NCBI’s Gene Expression Omnibus (GEO) under accession GSE217067.

RPKM data were log_2_ and median centered in R prior to data being visualized via Cluster 3.0 (ref. 21; http://bonsai.hgc.jp/~mdehoon/software/cluster/software.htm) and Java Treeview (ref. 22; https://jtreeview.sourceforge.net/). Pathway enrichment in gene lists was determined using g:Profiler. Data visualization for subtype progression between baseline through month 6 was assessed for statistical significance by Fisher’s exact test for each molecular subtype.

### GSEA.

To identify differentially enriched molecular pathways, GSEA was run as a Gene Pattern module (https://www.genepattern.org/#gsc.tab=0) using the gene set permutation option (23). The C2:Reactome gene set database from Molecular Signatures Database was used (v6.1 MSigDB) (24, 25). GSEA was performed on various comparisons and only results with a FDR of less than 10% were reported. The GSEA.html output files defined core enrichment.

### Statistics.

Comparison of the gene expression of the Costimulation of the CD28 Family pathway in all trial participants and the inflammatory subset–only trial participants at baseline versus month 6 was assessed using 2-tailed paired *t* test, with significance set at *P* < 0.05. Baseline gene expression of the Costimulation of the CD28 Family pathway among molecular subsets was compared using a 1-way ANOVA plus Tukey’s test for multiple comparisons, with significance set at *P* < 0.05. Association of the Costimulation of the CD28 Family pathway at baseline with change in mRSS (between baseline and 12 months) was conducted using Pearson’s correlation, with *P* < 0.05. All statistical tests were performed using R v3.4.3 using the “stats” package in baseR.

### Study approval.

Written informed consent was obtained from each participant. Each participating site’s institutional review board or ethics committee approved the study protocol for the clinical trial before the research commenced (10). The participating sites included Arthritis Associates of Southern California, Los Angeles, California, USA; UCLA, Los Angeles, California, USA; Stanford University, Redwood City, California, USA; Georgetown University, Washington, DC, USA; Northwestern University, Chicago, Illinois, USA; Harvard Mass General, Boston, Massachusetts, USA; Boston University, Boston, Massachusetts, USA; University of Michigan, Ann Arbor, Michigan, USA; University of Minnesota, Minneapolis, Minnesota, USA; Mayo Clinic, Rochester, Minnesota, USA; Rutgers University Clinical Research Center, New Brunswick, New Jersey, USA; Steffens Scleroderma Center, Albany, New York, USA; NorthWell Health, Great Neck, New York, USA; Hospital for Special Surgery, New York, New York, USA; Columbia University, New York, New York, USA; Cleveland Clinic, Cleveland, Ohio, USA;Ohio State University Medical Center, Columbus, Ohio, USA; University of Pennsylvania, Philadelphia, Pennsylvania, USA; University of Pittsburgh, Pittsburgh, Pennsylvania, USA; Medical University of South Carolina, Charleston, South Carolina, USA; University of Texas Health Center at Houston, Houston, Texas, USA; University of Utah, Salt Lake City, Utah, USA; Swedish Health Services, Seattle, Washington, USA; St. Joseph Health Care London, London, Ontario, Canada; Mount Sinai Hospital, Toronto, Ontario, Canada; Jewish General Hospital, Montreal, Quebec, Canada; and Royal Free Hospital, London, United Kingdom (ClinicalTrials.gov NCT02161406). The Dartmouth College Committee for the Protection of Human Subjects (Hanover, New Hampshire) reviewed and approved all protocols related to the gene expression analyses.

## Author contributions

MLW, DK, DAF, and CS designed the research study. BKM, JMF, YY, YW, and TW conducted experiments. BKM, TW, DK, and MLW acquired data. BKM, MEE, JMF, YY, YW, TW, JEG, CS, DAF, DK, and MLW analyzed data. BKM, MEE, DK, and MLW wrote the manuscript. All authors approved the manuscript. BKM and MEE are co–first authors. BKM is listed first because BKM performed all initial RNA-Seq of samples and bioinformatic analyses and drafted the manuscript. MEE completed the bioinformatic analyses and the manuscript.

## Supplementary Material

Supplemental data

## Figures and Tables

**Table 1:**
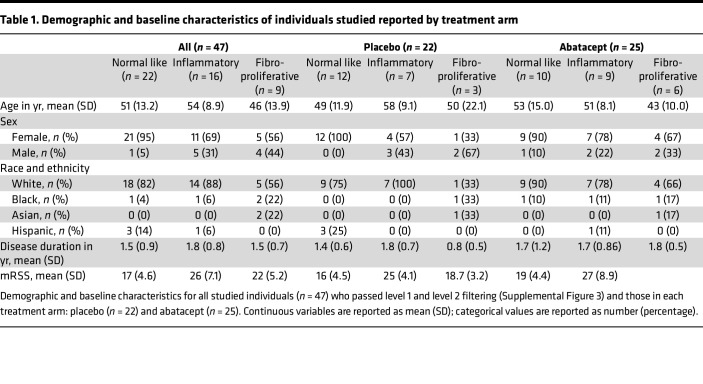
Demographic and baseline characteristics of individuals studied reported by treatment arm

**Table 2:**
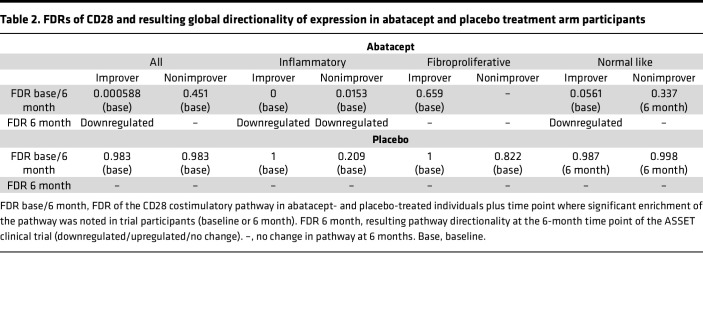
FDRs of CD28 and resulting global directionality of expression in abatacept and placebo treatment arm participants

## Version 1 11/10/2022

In-Press Preview

## Version 2 12/22/2022

Electronic publication
