# Supplementary material for: Machine-learning classification identifies patients with early systemic sclerosis as abatacept responders via CD28 pathway modulation
Source: JCI Insight. 2022 Dec 22;7(24):e155282. doi: 10.1172/jci.insight.155282 (PMC9869963; doi:10.1172/jci.insight.155282)
Supplement: Supplemental data [file jciinsight-7-155282-s151.pdf]

Supplement:

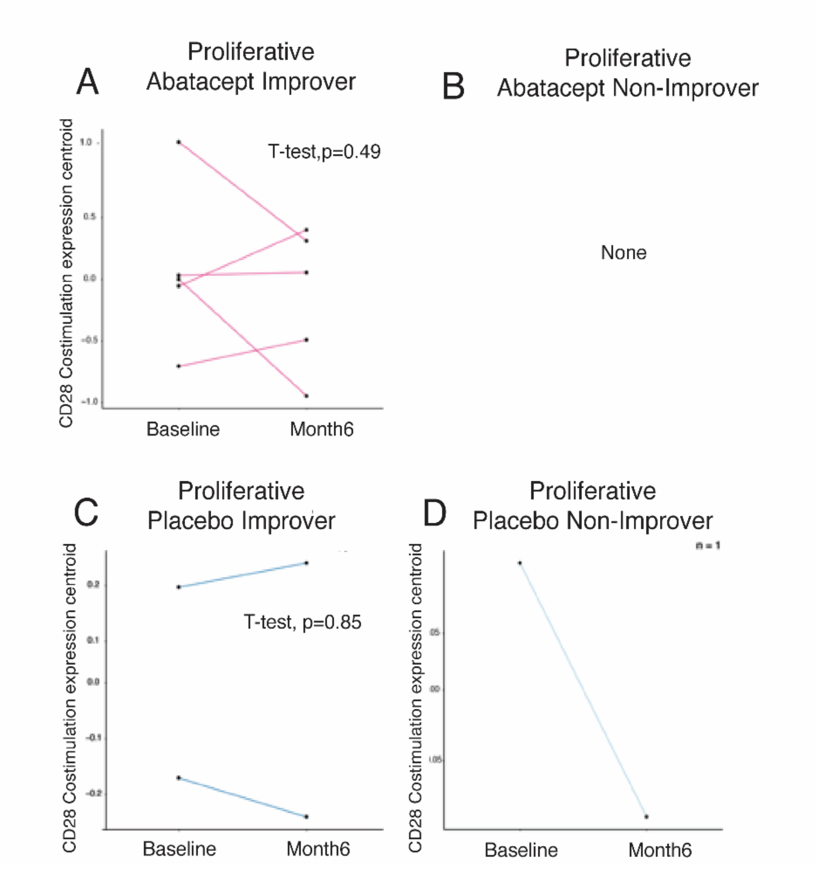

Supplementary Figure 1: Comparing *Costimulation of CD28 family* average expression for patients in the proliferative subset between baseline and 6-month time points. A. Average gene expression for core enrichment genes in *Costimulation of CD28 family* pathway in proliferative patients on abatacept that improved. Data is log2 and median centered. B. Average gene expression for core enrichment genes in *Costimulation of CD28 family* pathway in proliferative patients on abatacept that did not improve. Data is log2 and median centered. C. Average gene expression for core enrichment genes in *Costimulation of CD28 family* pathway in proliferative patients on placebo that improved. Data is log2 and median centered. D. Average gene expression for core enrichment genes in *Costimulation of CD28 family* pathway in

proliferative patients on placebo that did not improve. Data is log2 and median centered. Paired T-test p-values shown.

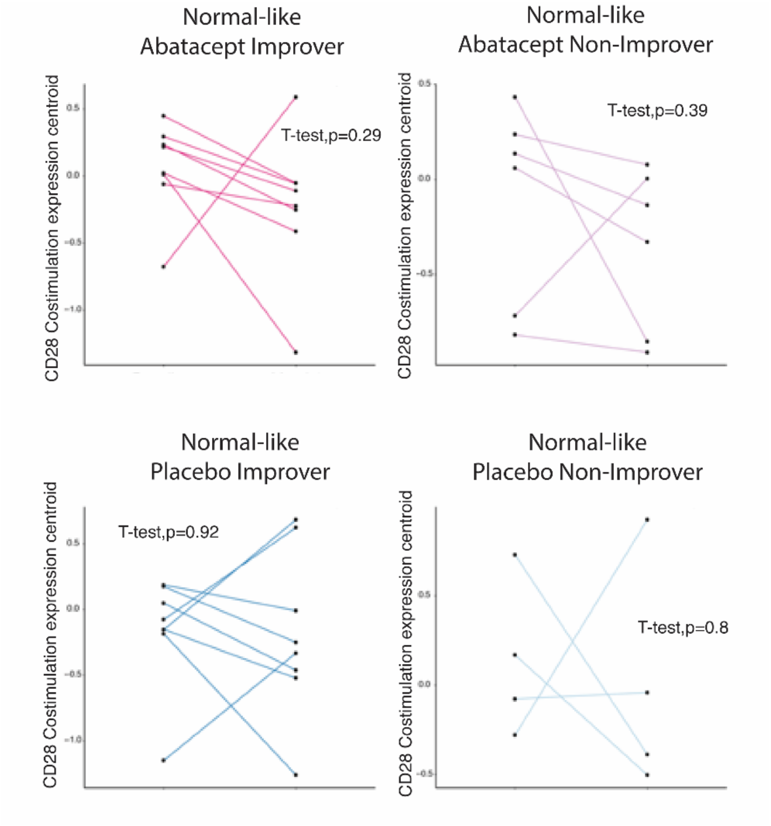

Supplementary Figure 2: Comparing *Costimulation of CD28 family* average expression for patients in the normal-like subset between baseline and 6-month time points. A. Average gene expression for core enrichment genes in *Costimulation of CD28 family* pathway in normal-like patients on abatacept that improved. Data is log2 and median centered. B. Average gene expression for core enrichment genes in *Costimulation of CD28 family* pathway in normal-like patients on abatacept that did not improve. Data is log2 and median centered. C. Average gene expression for core enrichment genes in *Costimulation of CD28 family* pathway in normal-like patients on placebo that improved. Data is log2 and median centered. D. Average gene expression for core

enrichment genes in *Costimulation of CD28 family* pathway in normal-like patients on placebo that did not improve. Data is log2 and median centered. Paired T-test pvalues shown.

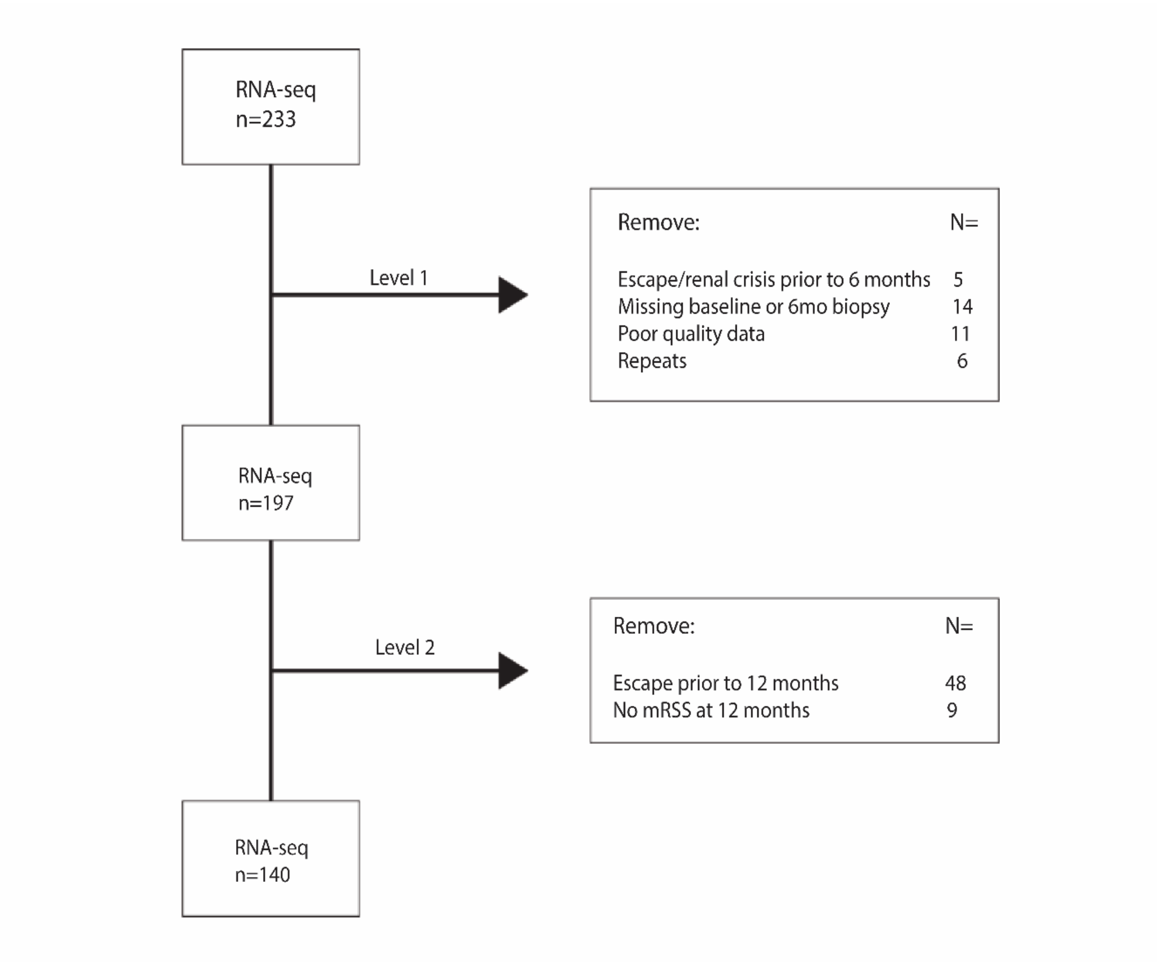

Supplementary Figure 3: Data cleaning prior to analyses at two exclusion levels based on data and patient related factors.

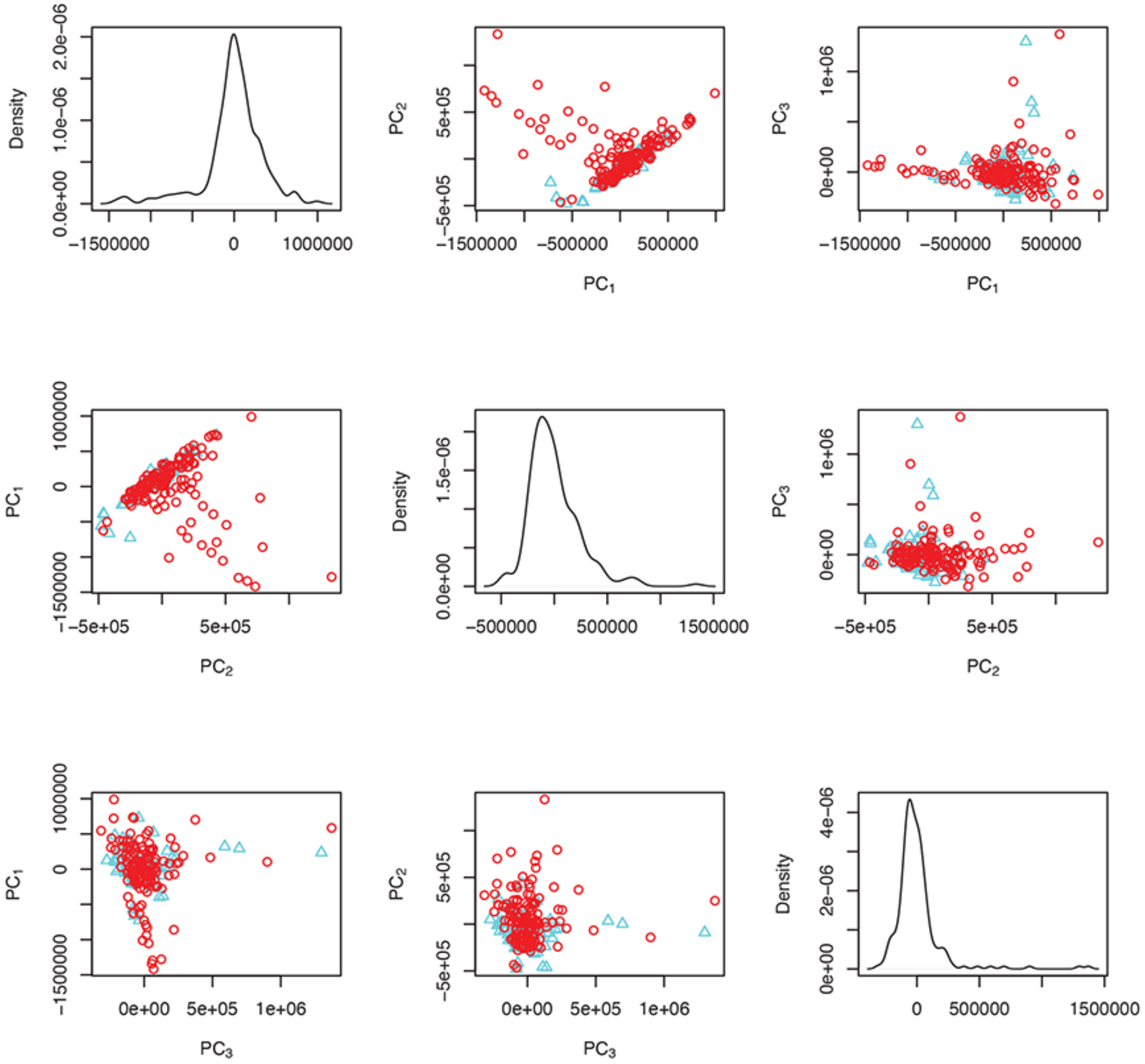

Supplementary Figure 4: PCA on raw RNA-seq counts for isolates included in this analysis (N=47); gPCA P-value =0.612.

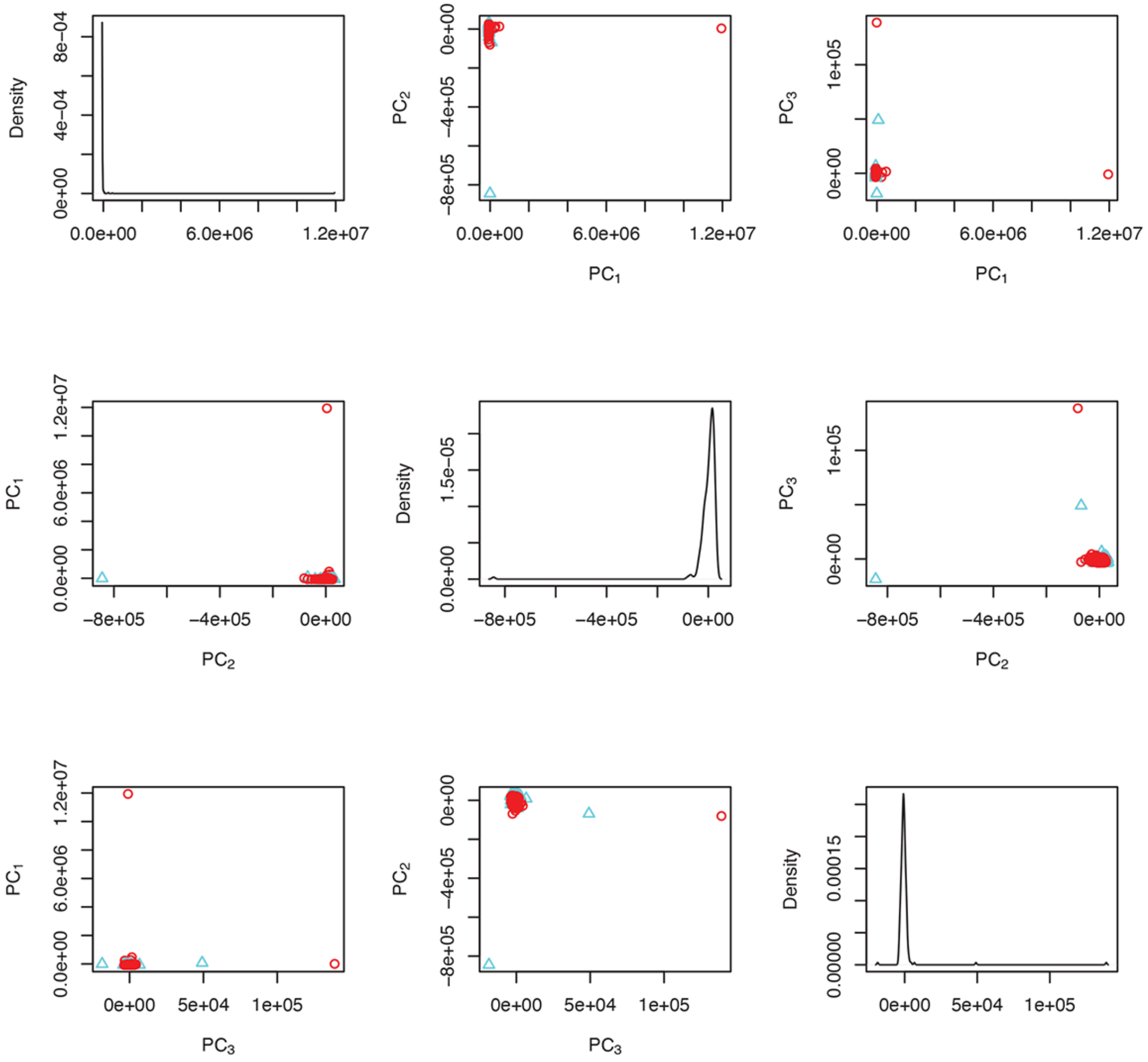

Supplementary Figure 5: PCA on RPKM normalized RNA-seq counts for isolates included in this analysis (N=47); gPCA P-value =0.594.

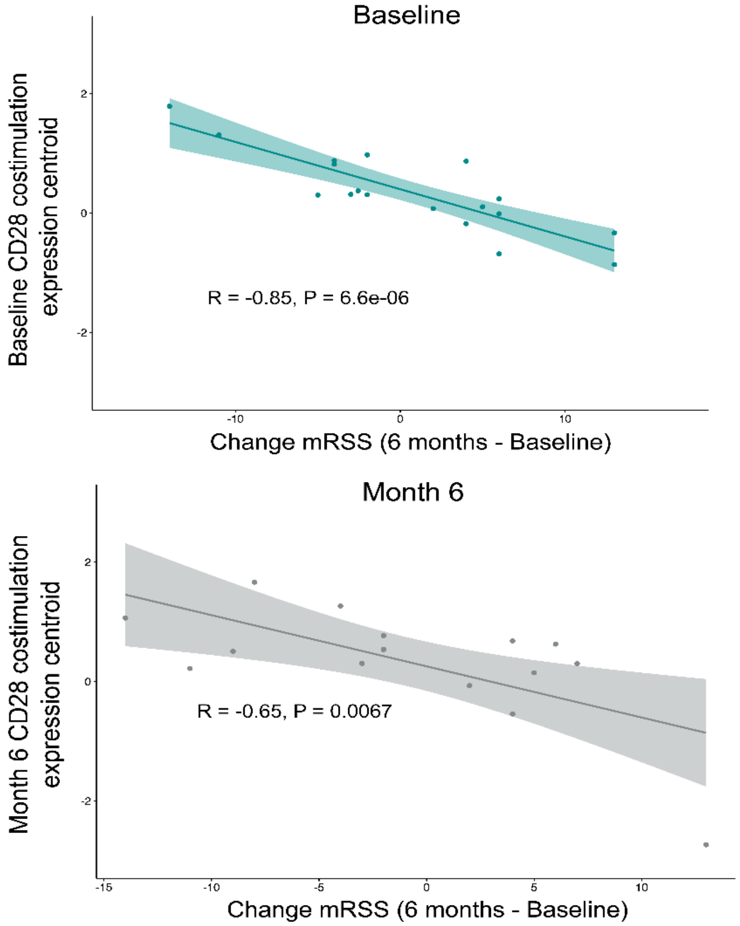

Supplementary Figure 6: Correlation of mRSS at month 6. Month 6 of treatment correlated with average expression of core genes in *Costimulation by CD28 Family* in Inflammatory individuals at Baseline (A) and Month 6 (B).

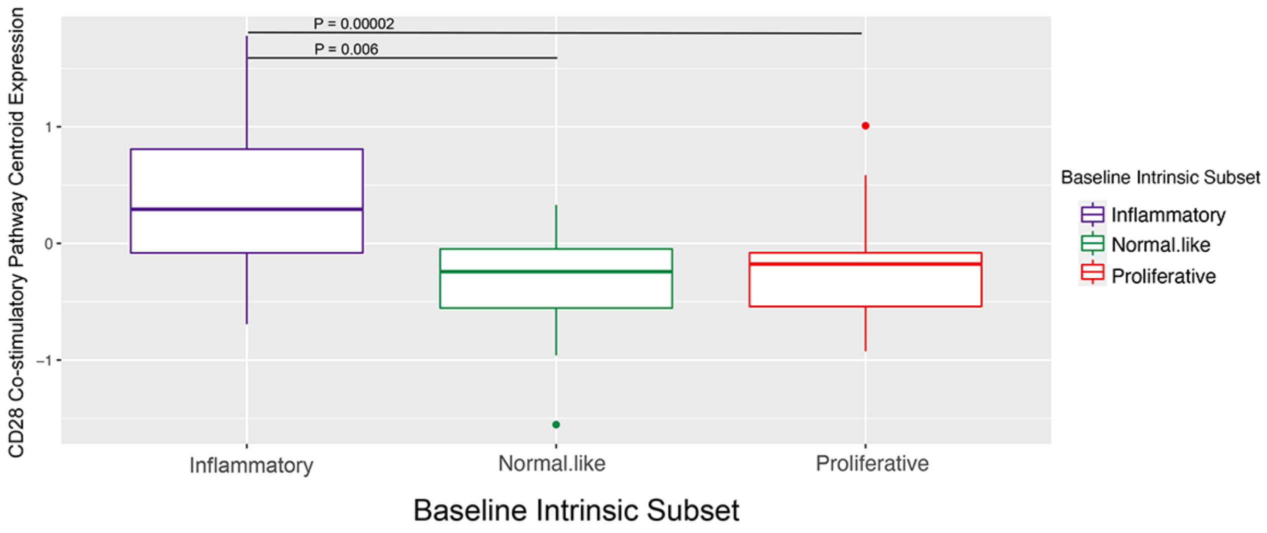

Supplementary Figure 7: CD28 Co-stimulatory pathway centroid expression for RNA-seq data, including data excluded according to Exclusion Criteria Level 2 by molecular subset. All individuals' RNA-seq data (N=67) included with no exclusionary criteria set. Statistical significance assessed via ANOVA plus Tukey's HSD test at P=0.05 significance threshold.

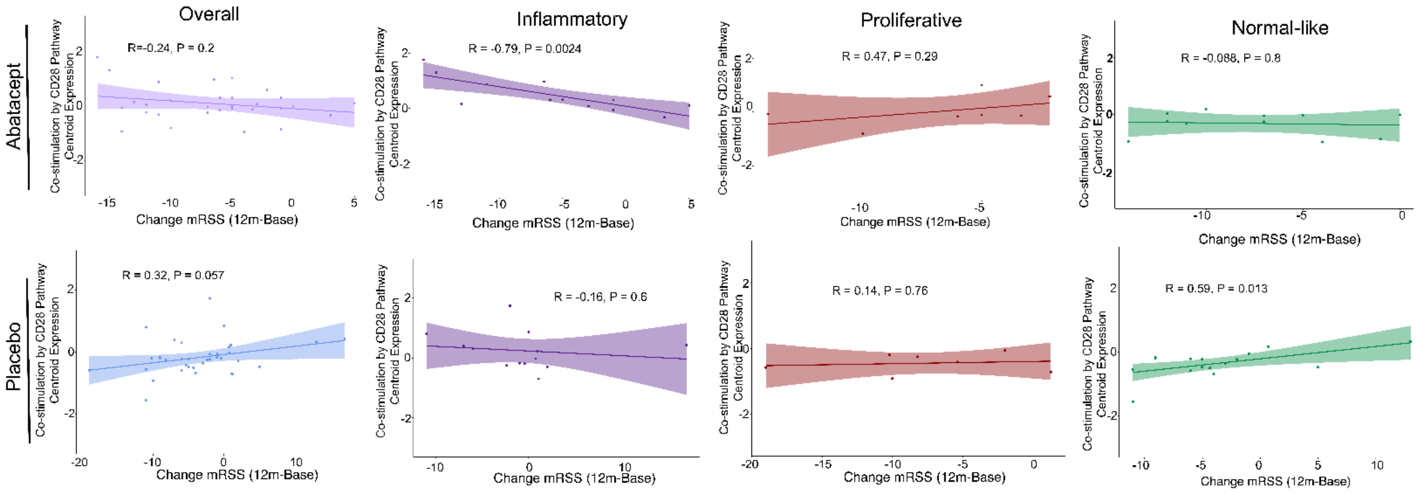

Supplementary Figure 8: Treatment-arm and molecular-subtype stratified correlation between CD28 pathway expression and change in mRSS (baseline to 12 months) for all baseline arrays (N=67). This includes those excluded due to Exclusionary Criteria Level 2 with corresponding confidence coefficient with significance assessed at P=0.05 threshold.

A

Abatacept Improvers

Reactome Terms Enriched in Base

Rank	NAME	NOM p-val	FDR q-val
1	IMMUNOREGULATORY_INTERACTIONS_BETWEEN_A_LYMPHOID_AND_A_NON_LYMPHOID_CELL	0	0
2	GENERATION_OF_SECOND_MESSENGER_MOLECULES	0	0
3	CHEMOKINE_RECEPTORS_BIND_CHEMOKINES	0	0
4	PD1_SIGNALING	0	2.76E-04
5	3_UTR_MEDIATED_TRANSLATIONAL_REGULATION	0	3.45E-04
6	PEPTIDE_CHAIN_ELONGATION	0	4.65E-04
7	*COSTIMULATION_BY_THE_CD28_FAMILY*	0	5.89E-04
8	RNA_POL_I_PROMOTER_OPENING	0	0.00191392
9	TCR_SIGNALING	0	0.00212901
10	DEPOSITION_OF_NEW_CENPA_CONTAINING_NUCLEOSOMES_AT_THE_CENTROMERE	0	0.00228832

Reactome Terms Enriched in 6 Month

Rank	NAME	NOM p-val	FDR q-val
1	COLLAGEN_FORMATION	0	0.04510721
2	EXTRACELLULAR_MATRIX_ORGANIZATION	0	0.07763565

B

Placebo Improvers

Reactome Terms Enriched in Base

Rank	NAME	NOM p-val	FDR q-val
1	G_ALPHA_S_SIGNALLING_EVENTS	0	0.00172411
2	GLUCOSE_METABOLISM	0	0.00427812
3	LIPID_DIGESTION_MOBILIZATION_AND_TRANSPORT	0	0.00285208
4	TRIGLYCERIDE_BIOSYNTHESIS	0	0.00706972
5	FATTY_ACID_TRIACYLGLYCEROL_AND_KETONE_BODY_METABOLISM	0	0.02970582
6	METABOLISM_OF_VITAMINS_AND_COFACTORS	0	0.02532122
7	PYRUVATE_METABOLISM_AND_CITRIC_ACID_TCA_CYCLE	0.00149254	0.02450977
8	GPCR_DOWNSTREAM_SIGNALING	0	0.0278584
9	AMINE_LIGAND_BINDING_RECEPTORS	0	0.03153476
10	SIGNALING_BY_FGFR1_MUTANTS	0.00321543	0.03867875

Reactome Terms Enriched in 6 Month

Rank	NAME	NOM p-val	FDR q-val
1	SRP_DEPENDENT_COTRANSLATIONAL_PROTEIN_TARGETING_TO_MEMBRANE	0	0
2	PEPTIDE_CHAIN_ELONGATION	0	0
3	3_UTR_MEDIATED_TRANSLATIONAL_REGULATION	0	0
4	TRANSLATION	0	0
5	NONSENSE_MEDIATED_DECAY_ENHANCED_BY_THE_EXON_JUNCTION_COMPLEX	0	0
6	INFLUENZA_VIRAL_RNA_TRANSCRIPTION_AND_REPLICATION	0	0
7	INFLUENZA_LIFE_CYCLE	0	0
8	METABOLISM_OF_MRNA	0	0
9	ACTIVATION_OF_THE_MRNA_UPON_BINDING_OF_THE_CAP_BINDING_COMPLEX_AND_EIFS_AND_SUBSEQUENT_BINDING_TO_43S	0	0
10	FORMATION_OF_THE_TERNARY_COMPLEX_AND_SUBSEQUENTLY_THE_43S_COMPLEX	0	0

C

Abatacept Non-Improvers

Reactome Terms Enriched in Base

Rank	NAME	NOM p-val	FDR q-val
1	PD1_SIGNALING	0	0.00254174
2	PEPTIDE_CHAIN_ELONGATION	0	0.00375343
3	PTM_GAMMA_CARBOXYLATION_HYPUSINE_FORMATION_AND_ARYLSULFATASE_ACTIVATION	0	0.00560049
4	INFLUENZA_VIRAL_RNA_TRANSCRIPTION_AND_REPLICATION	0	0.00638251
5	GLYCOSPHINGOLIPID_METABOLISM	0	0.00672876
6	POST_CHAPERONIN_TUBULIN_FOLDING_PATHWAY	0	0.00678962
7	3_UTR_MEDIATED_TRANSLATIONAL_REGULATION	0	0.00742545
8	FORMATION_OF_TUBULIN_FOLDING_INTERMEDIATES_BY_CCT_TRIC	0	0.00875328
9	SRP_DEPENDENT_COTRANSLATIONAL_PROTEIN_TARGETING_TO_MEMBRANE	0	0.00966214
10	PREFOLDIN_MEDIATED_TRANSFER_OF_SUBSTRATE_TO_CCT_TRIC	0.00412371	0.0171055

Reactome Terms Enriched in 6 Month

Rank	NAME	NOM p-val	FDR q-val
1	GENERIC_TRANSCRIPTION_PATHWAY	0	8.14E-04
2	TRIGLYCERIDE_BIOSYNTHESIS	0	0.10085949
3	SIGNALING_BY_FGFR_MUTANTS	0.00186916	0.17371553
4	G_ALPHA_S_SIGNALLING_EVENTS	0.00746269	0.22455192
5	FATTY_ACYL_COA_BIOSYNTHESIS	0.0038835	0.22479491
6	POST_NMDA_RECEPTOR_ACTIVATION_EVENTS	0.00784314	0.23800428
7	SIGNALING_BY_FGFR1_FUSION_MUTANTS	0.01188119	0.24183348
8	BRANCHED_CHAIN_AMINO_ACID_CATABOLISM	0.0155642	0.24825512
9	FATTY_ACID_TRIACYLGLYCEROL_AND_KETONE_BODY_METABOLISM	0	0.26131243
10	PEROXISOMAL_LIPID_METABOLISM	0.01529637	0.28123292

D

Placebo Non-Improvers

Reactome Terms Enriched in Base

Rank	NAME	NOM p-val	FDR q-val
1	TCR_SIGNALING	0	0.00300951
2	DOWNSTREAM_TCR_SIGNALING	0	0.01881216
3	RORA_ACTIVATES_CIRCADIAN_EXPRESSION	0.00200401	0.02475553
4	GENERATION_OF_SECOND_MESSENGER_MOLECULES	0	0.03107577
5	FACTORS_INVOLVED_IN_MEGAKARYOCYTE_DEVELOPMENT_AND_PLATELET_PRODUCTION	0	0.0324196
6	YAP1_AND_WWTR1_TAZ_STIMULATED_GENE_EXPRESSION	0	0.03273465
7	CIRCADIAN_REPRESSION_OF_EXPRESSION_BY_REV_ERBA	0	0.04623171
8	ANTIGEN_ACTIVATES_B_CELL_RECEPTOR_LEADING_TO_GENERATION_OF_SECOND_MESSENGERS	0.00208333	0.06987141
9	PD1_SIGNALING	0.00603622	0.07585082
10	PHOSPHORYLATION_OF_CD3_AND_TCR_ZETA_CHAINS	0	0.07866955

Reactome Terms Enriched in 6 Month

Rank	NAME	NOM p-val	FDR q-val
1	RESPIRATORY_ELECTRON_TRANSPORT	0	0
2	RESPIRATORY_ELECTRON_TRANSPORT_ATP_SYNTHESIS_BY_CHEMIOSMOTIC_COUPLING_AND_HEAT_PRODUCTION_BY_UNCOUPL	0	0
3	TCA_CYCLE_AND_RESPIRATORY_ELECTRON_TRANSPORT	0	0
4	SRP_DEPENDENT_COTRANSLATIONAL_PROTEIN_TARGETING_TO_MEMBRANE	0	0
5	TRANSLATION	0	0
6	TRNA_AMINOACYLATION	0	0
7	PEPTIDE_CHAIN_ELONGATION	0	0
8	METABOLISM_OF_PROTEINS	0	0
9	CYTOSOLIC_TRNA_AMINOACYLATION	0	9.60E-05
10	3_UTR_MEDIATED_TRANSLATIONAL_REGULATION	0	1.06E-04

Supplementary Table 1. GSEA between base and 6-month time points stratified by treatment arm and improvement status. A. Patients that improved on abatacept, showing top 10 (<FDR 10%) pathways enriched in base and 6-month time points. B. Patients that improved on Placebo, showing top 10 (<FDR 10%) pathways enriched in base and 6-month time points. C. Patients that did not improve on abatacept, showing top 10 (<FDR 10%) pathways enriched in base and 6-month time points. D. Patients that did not improve on Placebo, showing top 10 (<FDR 10%) pathways enriched in base and 6-month time points.

A

Inflammatory Abatacept Improvers

Reactome Terms Enriched in Base

Rank	NAME	NOM p-val	FDR q-val
1	*IMMUNOREGULATORY_INTERACTIONS_BETWEEN_A_LYMPHOID_AND_A_NON_LYMPHOID_CELL*	0	0
2	GENERATION_OF_SECOND_MESSENGER_MOLECULES	0	0
3	CHEMOKINE_RECEPTORS_BIND_CHEMOKINES	0	0
4	*COSTIMULATION_BY_THE_CD28_FAMILY*	0	0
5	*INTERFERON_ALPHA_BETA_SIGNALING*	0	0
6	*PD1_SIGNALING*	0	0
7	INTERFERON_GAMMA_SIGNALING	0	0
8	CYTOKINE_SIGNALING_IN_IMMUNE_SYSTEM	0	0
9	*TCR_SIGNALING*	0	0
10	G_ALPHA_I_SIGNALLING_EVENTS	0	0

Reactome Terms Enriched in 6 Month

Rank	NAME	NOM p-val	FDR q-val
1	COLLAGEN_FORMATION	0	0.0188875
2	RESPIRATORY_ELECTRON_TRANSPORT	0	0.02069414
3	BRANCHED_CHAIN_AMINO_ACID_CATABOLISM	0.00183824	0.08063205
4	GENERIC_TRANSCRIPTION_PATHWAY	0	0.09051869

B

Inflammatory Placebo Improvers

Reactome Terms Enriched in Base

Rank	NAME	NOM p-val	FDR q-val
1	PEPTIDE_CHAIN_ELONGATION	0	0.03227363
2	COMPLEMENT_CASCADE	0.00166667	0.03275805
3	TRANSPORT_TO_THE_GOLGI_AND_SUBSEQUENT_MODIFICATION	0.00321027	0.05267023
4	HS_GAG_BIOSYNTHESIS	0.00331675	0.05657585
5	GABA_RECEPTOR_ACTIVATION	0.00160772	0.05719304
6	N_GLYCAN_ANTENNAE_ELONGATION_IN_THE_MEDIAL_TRANS_GOLGI	0.00338983	0.06000441
7	INWARDLY_RECTIFYING_K_CHANNELS	0.0049505	0.06047099
8	LIPOPROTEIN_METABOLISM	0	0.06123978
9	SRP_DEPENDENT_COTRANSLATIONAL_PROTEIN_TARGETING_TO_MEMBRANE	0	0.06194396
10	CLASS_A1_RHODOPSIN_LIKE_RECEPTORS	0	0.06295773

Reactome Terms Enriched in 6 Month

Rank	NAME	NOM p-val	FDR q-val
1	MRNA_3_END_PROCESSING	0	0.0241332
2	MRNA_SPLICING	0	0.03424016
3	PROCESSING_OF_CAPPED_INTRON_CONTAINING_PRE_MRNA	0	0.03714498
4	INTERFERON_ALPHA_BETA_SIGNALING	0	0.04367536
5	RESOLUTION_OF_AP_SITES_VIA_THE_MULTIPLE_NUCLEOTIDE_PATCH_REPLACEMENT_PATHWAY	0.00234742	0.04541393
6	BASE_EXCISION_REPAIR	0.00273224	0.04803406
7	CLEAVAGE_OF_GROWING_TRANSCRIPT_IN_THE_TERMINATION_REGION_	0.00802139	0.0795131
8	MITOTIC_M_M_G1_PHASES	0	0.08853283
9	DNA_REPLICATION	0	0.08951623

C

Inflammatory Abatacept Non-Improvers

Reactome Terms Enriched in Base

Rank	NAME	NOM p-val	FDR q-val
1	*IMMUNOREGULATORY_INTERACTIONS_BETWEEN_A_LYMPHOID_AND_A_NON_LYMPHOID_CELL*	0	0
2	EXTRACELLULAR_MATRIX_ORGANIZATION	0	0
3	CHONDROITIN_SULFATE_BIOSYNTHESIS	0	0.00220795
4	DEGRADATION_OF_THE_EXTRACELLULAR_MATRIX	0	0.00275994
5	PD1_SIGNALING	0	0.00367992
6	NCAM1_INTERACTIONS	0	0.00382566
7	COLLAGEN_FORMATION	0	0.00751638
8	CHONDROITIN_SULFATE_DERMATAN_SULFATE_METABOLISM	0.00167504	0.01284466
9	CELL_SURFACE_INTERACTIONS_AT_THE_VASCULAR_WALL	0	0.01362603
10	INTEGRIN_CELL_SURFACE_INTERACTIONS	0	0.01393508
		0	0.01533159
14	*COSTIMULATION_BY_THE_CD28_FAMILY*		

Reactome Terms Enriched in 6 Month

Rank	NAME	NOM p-val	FDR q-val
1	G2_M_CHECKPOINTS	0	0.00290258
2	PEPTIDE_CHAIN_ELONGATION	0	0.04308269
3	MITOTIC_PROMETAPHASE	0	0.04319526
4	ACTIVATION_OF_ATR_IN_RESPONSE_TO_REPLICATION_STRESS	0	0.04591967
5	MITOTIC_M_M_G1_PHASES	0	0.04797228
6	CELL_CYCLE_MITOTIC	0	0.05162922
7	DNA_REPLICATION	0	0.05164407
8	MITOTIC_G2_G2_M_PHASES	0	0.05245843
9	DEPOSITION_OF_NEW_CENPA_CONTAINING_NUCLEOSOMES_AT_THE_CENTROMERE	0.00255102	0.05258824
10	BRANCHED_CHAIN_AMINO_ACID_CATABOLISM	0.00437637	0.05549848

D

Inflammatory Placebo Non-Improvers

Reactome Terms Enriched in Base

Rank	NAME	NOM p-val	FDR q-val
1	G_BETA_GAMMA_SIGNALLING_THROUGH_PLC_BETA	0.00194932	0.06028763
2	THROMBIN_SIGNALLING_THROUGH_PROTEINASE_ACTIVATED_RECEPTORS_PARS	0.00922509	0.06086005
3	YAP1_AND_WWTR1_TAZ_STIMULATED_GENE_EXPRESSION	0.00576923	0.06540132
4	EFFECTS_OF_PIP2_HYDROLYSIS	0.00766284	0.06577846
5	INHIBITION_OF_INSULIN_SECRETION_BY_ADRENALINE_NORADRENALINE	0.00369004	0.07058195
6	OLFACTORY_SIGNALING_PATHWAY	0.00733945	0.07394856
7	VOLTAGE_GATED_POTASSIUM_CHANNELS	0.00369004	0.07481439
8	RORA_ACTIVATES_CIRCADIAN_EXPRESSION	0.0094697	0.08254773
9	CIRCADIAN_REPRESSION_OF_EXPRESSION_BY_REV_ERBA	0.01351351	0.08530546
10	TRANSPORT_OF_INORGANIC_CATIONS_ANIONS_AND_AMINO_ACIDS_OLIGOPEPTIDES	0.00185874	0.08923493

Reactome Terms Enriched in 6 Month

Rank	NAME	NOM p-val	FDR q-val
1	RESPIRATORY_ELECTRON_TRANSPORT	0	0
2	RESPIRATORY_ELECTRON_TRANSPORT_ATP_SYNTHESIS_BY_CHEMIOSMOTIC_COUPLING_AND_HEAT_PRODUCTION_BY_UNCOUPL	0	0
3	TRANSLATION	0	0
4	SRP_DEPENDENT_COTRANSLATIONAL_PROTEIN_TARGETING_TO_MEMBRANE	0	0
5	3_UTR_MEDIATED_TRANSLATIONAL_REGULATION	0	0
6	TCA_CYCLE_AND_RESPIRATORY_ELECTRON_TRANSPORT	0	0
7	PEPTIDE_CHAIN_ELONGATION	0	0
8	METABOLISM_OF_RNA	0	0
9	METABOLISM_OF_PROTEINS	0	0
10	NONSENSE_MEDIATED_DECAY_ENHANCED_BY_THE_EXON_JUNCTION_COMPLEX	0	0

Supplementary Table 2.

GSEA between base and 6-month time points of inflammatory patients stratified by treatment arm and improvement status. A. Patients that improved on abatacept, showing top 10 (<FDR 10%) pathways enriched in base and 6-month time points. **B.** Patients that improved on Placebo, showing top 10 (<FDR 10%) pathways enriched in base and 6-month time points. **C.** Patients that did not improve on abatacept, showing top 10 (<FDR 10%) pathways enriched in base and 6-month time points. **D.** Patients that did not improve on Placebo, showing top 10 (<FDR 10%) pathways enriched in base and 6-month time points.

A

Proliferative Abatacept Improvers

Reactome Terms Enriched in Base

Rank	NAME	NOM p-val	FDR q-val
1	STRIATED_MUSCLE_CONTRACTION	0	0
2	CHOLESTEROL_BIOSYNTHESIS	0	0
3	MUSCLE_CONTRACTION	0	0.00259936
4	CHEMOKINE_RECEPTORS_BIND_CHEMOKINES	0	0.0077599
5	TRIGLYCERIDE_BIOSYNTHESIS	0	0.02040501
6	GLUCONEOGENESIS	0.00197239	0.04369375
7	PEPTIDE_LIGAND_BINDING_RECEPTORS	0	0.09510595

Reactome Terms Enriched in 6 Month

Rank	NAME	NOM p-val	FDR q-val
	None under 10% FDR		

B

Proliferative Placebo Improvers

Reactome Terms Enriched in Base

Rank	NAME	NOM p-val	FDR q-val
1	RNA_POL_I_PROMOTER_OPENING	0	0.00161192
2	AMYLOIDS	0	0.0493639
3	TIGHT_JUNCTION_INTERACTIONS	0.0018315	0.08159453

Reactome Terms Enriched in 6 Month

Rank	NAME	NOM p-val	FDR q-val
	None under 10% FDR		

C

Proliferative Abatacept Non-Improvers

No samples were both Proliferative at Baseline and Non-improvers

D

Proliferative Placebo Non-Improvers

Reactome Terms Enriched in Base

Rank	NAME	NOM p-val	FDR q-val
	None under 10% FDR		

Reactome Terms Enriched in 6 Month

Rank	NAME	NOM p-val	FDR q-val
1	RESPIRATORY_ELECTRON_TRANSPORT	0	0.07998024

Supplementary Table 3.

GSEA between base and 6-month time points of proliferative patients stratified by treatment arm and improvement status. A. Patients that improved on abatacept, showing top 10 (<FDR 10%) pathways enriched in base and 6-month time points. **B.** Patients that improved on Placebo, showing top 10 (<FDR 10%) pathways enriched in base and 6-month time points. **C.** Patients that did not improve on abatacept, showing top 10 (<FDR 10%) pathways enriched in base and 6-

month time points. **D.** Patients that did not improve on placebo, showing top 10 (<FDR 10%) pathways enriched in base and 6-month time points.

A

Normal-like Abatacept Improvers

Reactome Terms Enriched in Base

Rank	NAME	NOM p-val	FDR q-val
1	PEPTIDE_CHAIN_ELONGATION	0	0
2	INFLUENZA_VIRAL_RNA_TRANSCRIPTION_AND_REPLICATION	0	0
3	RESPIRATORY_ELECTRON_TRANSPORT	0	0
4	3_UTR_MEDIATED_TRANSLATIONAL_REGULATION	0	0
5	SRP_DEPENDENT_COTRANSLATIONAL_PROTEIN_TARGETING_TO_MEMBRANE	0	0
6	RESPIRATORY_ELECTRON_TRANSPORT_ATP_SYNTHESIS_BY_CHEMIOSMOTIC_COUPLING_AND_HEAT_PRODUCTION_BY_UNCOUPL	0	0
7	INFLUENZA_LIFE_CYCLE	0	0
8	NONSENSE_MEDIATED_DECAY_ENHANCED_BY_THE_EXON_JUNCTION_COMPLEX	0	0
9	TCA_CYCLE_AND_RESPIRATORY_ELECTRON_TRANSPORT	0	0
10	TRANSLATION	0	0

Reactome Terms Enriched in 6 Month

Rank	NAME	NOM p-val	FDR q-val
	None under 10% FDR		

B

Normal-like Placebo Improvers

Reactome Terms Enriched in Base

Rank	NAME	NOM p-val	FDR q-val
1	G_ALPHA_S_SIGNALLING_EVENTS	0	0.01779425
2	PYRUVATE_METABOLISM_AND_CITRIC_ACID_TCA_CYCLE	0	0.0181513
3	PLATELET_AGGREGATION_PLUG_FORMATION	0	0.01970872
4	AMINE_LIGAND_BINDING_RECEPTORS	0	0.0226796
5	GLUCOSE_METABOLISM	0	0.02712561
6	LIPID_DIGESTION_MOBILIZATION_AND_TRANSPORT	0	0.03654359
7	FATTY_ACYL_COA_BIOSYNTHESIS	0.0018315	0.03688335
8	TRIGLYCERIDE_BIOSYNTHESIS	0	0.04040688
9	GPCR_DOWNSTREAM_SIGNALING	0	0.05083709
10	CITRIC_ACID_CYCLE_TCA_CYCLE	0.00536673	0.05465682

Reactome Terms Enriched in 6 Month

Rank	NAME	NOM p-val	FDR q-val
1	NONSENSE_MEDIATED_DECAY_ENHANCED_BY_THE_EXON_JUNCTION_COMPLEX	0	0
2	PEPTIDE_CHAIN_ELONGATION	0	0
3	SRP_DEPENDENT_COTRANSLATIONAL_PROTEIN_TARGETING_TO_MEMBRANE	0	0
4	3_UTR_MEDIATED_TRANSLATIONAL_REGULATION	0	0
5	TRANSLATION	0	0
6	INFLUENZA_VIRAL_RNA_TRANSCRIPTION_AND_REPLICATION	0	0
7	INFLUENZA_LIFE_CYCLE	0	0
8	ACTIVATION_OF_THE_MRNA_UPON_BINDING_OF_THE_CAP_BINDING_COMPLEX_AND_EIFS_AND_SUBSEQUENT_BINDING_TO_43S	0	0
9	METABOLISM_OF_MRNA	0	0
10	FORMATION_OF_THE_TERNARY_COMPLEX_AND_SUBSEQUENTLY_THE_43S_COMPLEX	0	0

C

Normal-like Abatacept Non-Improvers

Reactome Terms Enriched in Base

Rank	NAME	NOM p-val	FDR q-val
1	PEPTIDE_CHAIN_ELONGATION	0	0
2	3_UTR_MEDIATED_TRANSLATIONAL_REGULATION	0	0
3	INFLUENZA_VIRAL_RNA_TRANSCRIPTION_AND_REPLICATION	0	0
4	NONSENSE_MEDIATED_DECAY_ENHANCED_BY_THE_EXON_JUNCTION_COMPLEX	0	0
5	SRP_DEPENDENT_COTRANSLATIONAL_PROTEIN_TARGETING_TO_MEMBRANE	0	0
6	TRANSLATION	0	0
7	INFLUENZA_LIFE_CYCLE	0	0
8	FORMATION_OF_THE_TERNARY_COMPLEX_AND_SUBSEQUENTLY_THE_43S_COMPLEX	0	0
9	ACTIVATION_OF_THE_MRNA_UPON_BINDING_OF_THE_CAP_BINDING_COMPLEX_AND_EIFS_AND_SUBSEQUENT_BINDING_TO_43S	0	0
10	METABOLISM_OF_MRNA		

Reactome Terms Enriched in 6 Month

Rank	NAME	NOM p-val	FDR q-val
1	G_ALPHA_S_SIGNALLING_EVENTS	0	0.01185296
2	SIGNALING_BY_FGFR1_FUSION_MUTANTS	0	0.01673589
3	GENERIC_TRANSCRIPTION_PATHWAY	0	0.01796875
4	TRIGLYCERIDE_BIOSYNTHESIS	0	0.02083848
5	ENERGY_DEPENDENT_REGULATION_OF_MTOR_BY_LKB1_AMPK	0	0.02802395
6	PYRUVATE_METABOLISM	0.00587084	0.02964978
7	CGMP_EFFECTS	0.00375235	0.04030581
8	NITRIC_OXIDE_STIMULATES_GUANYLATE_CYCLASE	0.0037594	0.04190546
9	GPCR_DOWNSTREAM_SIGNALING	0	0.0432813
10	SIGNALING_BY_FGFR1_MUTANTS	0	0.05050794

D

Normal-like Placebo Non-Improvers

Reactome Terms Enriched in Base

Rank	NAME	NOM p-val	FDR q-val
1	MEIOTIC_SYNAPSIS	0	3.75E-04
2	MEIOSIS	0	4.37E-04
3	RNA_POL_I_PROMOTER_OPENING	0	4.88E-04
4	MITOTIC_PROMETAPHASE	0	5.24E-04
5	MEIOTIC_RECOMBINATION	0	5.72E-04
6	CELL_CYCLE	0	6.56E-04
7	CHROMOSOME_MAINTENANCE	0	6.96E-04
8	RNA_POL_I_TRANSCRIPTION	0	7.54E-04
9	G2_M_CHECKPOINTS	0	7.74E-04
10	CELL_CYCLE_MITOTIC	0	8.22E-04

Reactome Terms Enriched in 6 Month

Rank	NAME	NOM p-val	FDR q-val
1	COLLAGEN_FORMATION	0	0.00902945
2	AMINE_LIGAND_BINDING_RECEPTORS	0	0.01113978
3	TRIGLYCERIDE_BIOSYNTHESIS	0	0.01352679
4	FGFR_LIGAND_BINDING_AND_ACTIVATION	0	0.01691916
5	G_ALPHA_S_SIGNALLING_EVENTS	0	0.01810906
6	FATTY_ACYL_COA_BIOSYNTHESIS	0.0094518	0.07568885
7	PLATELET_HOMEOSTASIS	0.00164204	0.08241602
8	EXTRACELLULAR_MATRIX_ORGANIZATION	0.00157233	0.08342381
9	FATTY_ACID_TRIACYLGLYCEROL_AND_KETONE_BODY_METABOLISM	0	0.0858466
10	NITRIC_OXIDE_STIMULATES_GUANYLATE_CYCLASE	0.00332779	0.08588468

Supplementary Table 4.

GSEA between base and 6-month time points of normal-like patients stratified by treatment arm and improvement status. A. Patients that improved on abatacept, showing top 10 (<FDR 10%) pathways enriched in base and 6-month time points. **B.** Patients that improved on Placebo, showing top 10 (<FDR 10%) pathways enriched in base and 6-month time points. **C.** Patients that did not improve on abatacept, showing top 10 (<FDR 10%) pathways enriched in base and 6-month time points. **D.** Patients that did not improve on Placebo, showing top 10 (<FDR 10%) pathways enriched in base and 6-month time points.

SSID	Baseline	Month3	Month6	Change	Treatment
02-0153	Inflammator	Inflammator	Inflammator	NO	Abatacept
08-1065	Inflammator	Inflammator	Inflammator	NO	Abatacept
05-0603	Inflammator	Inflammator	Inflammator	NO	Placebo
10-1353	Inflammator	Inflammator	Inflammator	NO	Placebo
16-2256	Inflammator	Inflammator	Inflammator	NO	Placebo
18-2553	Inflammator	Inflammator	Inflammator	NO	Placebo
11-1503	Inflammator	Inflammator	Normal.like	YES	Abatacept
20-2853	Inflammator	Inflammator	Normal.like	YES	Abatacept
30-4356	Inflammator	Inflammator	Normal.like	YES	Abatacept
30-4359	Inflammator	Inflammator	Normal.like	YES	Abatacept
31-4152	Inflammator	Inflammator	Normal.like	YES	Abatacept
40-5853	Inflammator	Inflammator	Proliferative	YES	Placebo
25-3609	Inflammator	Normal.like	Inflammator	YES	Placebo
01-0045	Inflammator	Proliferative	Inflammator	YES	Abatacept
01-0060	Inflammator	Proliferative	Inflammator	YES	Placebo
01-0003	Inflammator	Proliferative	Proliferative	YES	Abatacept
03-0312	Normal.like	NA	Normal.like	NO	Abatacept
26-3753	Normal.like	Normal.like	Inflammator	YES	Abatacept
08-1071	Normal.like	Normal.like	Inflammator	YES	Placebo
14-1956	Normal.like	Normal.like	Inflammator	YES	Placebo
01-0069	Normal.like	Normal.like	Normal.like	NO	Abatacept
06-0756	Normal.like	Normal.like	Normal.like	NO	Abatacept
11-1515	Normal.like	Normal.like	Normal.like	NO	Abatacept
17-2415	Normal.like	Normal.like	Normal.like	NO	Abatacept
30-4353	Normal.like	Normal.like	Normal.like	NO	Abatacept
31-4506	Normal.like	Normal.like	Normal.like	NO	Abatacept
01-0006	Normal.like	Normal.like	Normal.like	NO	Placebo
01-0018	Normal.like	Normal.like	Normal.like	NO	Placebo
01-0024	Normal.like	Normal.like	Normal.like	NO	Placebo
03-0327	Normal.like	Normal.like	Normal.like	NO	Placebo
04-0459	Normal.like	Normal.like	Normal.like	NO	Placebo
04-0462	Normal.like	Normal.like	Normal.like	NO	Placebo
08-1053	Normal.like	Normal.like	Normal.like	NO	Placebo
31-4515	Normal.like	Normal.like	Normal.like	NO	Placebo
40-5859	Normal.like	Normal.like	Normal.like	NO	Placebo
15-2103	Normal.like	Normal.like	Proliferative	YES	Placebo
08-1068	Normal.like	Proliferative	Proliferative	YES	Abatacept
31-4503	Normal.like	Proliferative	Proliferative	YES	Abatacept
01-0012	Proliferative	Normal.like	Normal.like	YES	Abatacept
17-2403	Proliferative	Normal.like	Normal.like	YES	Abatacept
25-3615	Proliferative	Normal.like	Normal.like	YES	Abatacept
03-0306	Proliferative	Proliferative	Inflammator	YES	Abatacept
01-0036	Proliferative	Proliferative	Normal.like	YES	Abatacept
09-1206	Proliferative	Proliferative	Proliferative	NO	Abatacept
01-0066	Proliferative	Proliferative	Proliferative	NO	Placebo
11-1509	Proliferative	Proliferative	Proliferative	NO	Placebo
31-4524	Proliferative	Proliferative	Proliferative	NO	Placebo

Change-Defined as change in intrinsic molecular subset by Month6

Supplementary Table 5.

Molecular Subtype Calls for Baseline through Month 6 for included patients (N=47).
